# Modified Immunoscore Improves Prediction of Survival Outcomes in Patients Undergoing Radical Cystectomy for Bladder Cancer—A Retrospective Digital Pathology Study

**DOI:** 10.3390/diagnostics12061360

**Published:** 2022-06-01

**Authors:** Uwe Bieri, Lorenz Buser, Marian Severin Wettstein, Daniel Eberli, Karim Saba, Holger Moch, Thomas Hermanns, Cédric Poyet

**Affiliations:** 1 Department of Urology, University Hospital of Zurich, University of Zurich, 8091 Zurich, Switzerland; uwe.bieri@usz.ch (U.B.); marianseverin.wettstein@usz.ch (M.S.W.); daniel.eberli@usz.ch (D.E.); sabakarim@gmail.com (K.S.); thomas.hermanns@usz.ch (T.H.); 2 Department of Pathology and Molecular Pathology, University Hospital of Zurich, University of Zurich, 8091 Zurich, Switzerland; lorenz.buser@unilabs.com (L.B.); holger.moch@usz.ch (H.M.)

**Keywords:** biomarker, bladder cancer, cystectomy, digital pathology, immunoscore

## Abstract

To evaluate the prognostic value of a modified Immunoscore (mIS) in a cohort of bladder cancer (BC) patients undergoing radical cystectomy (RC), two tissue microarrays of 159 BC patients were immunohistochemically stained for CD3/CD8/FOXP3 and CD45RO to detect Tumor-Infiltrating Lymphocytes (TIL). To predict progression free survival (PFS) and cancer specific survival (CSS), a predictive model cumulatively incorporating all four components was constructed and labeled as mIS. Patients were stratified into two risk groups; “high mIS/favorable risk” and “low mIS/unfavorable risk”. Kaplan–Meier analysis was used to test mIS within each American Joint Committee on Cancer (AJCC) stage group for BC. In a univariable cox regression analysis all single components used for mIS, showed a significant association with CSS. Patients with high mIS (all components) in the AJCC stage IIIa group additionally showed a significantly longer PFS (Hazard Ratio (HR): 2.7; *p* = 0.008) and CSS (HR: 3.5; *p* = 0.006) as compared to patients with low mIS. mIS is of prognostic value in BC patients undergoing RC and was able to stratify patients within AJCC stage IIIa and might thus serve as a prognostic marker to guide risk-adapted treatment or follow-up strategies after RC.

## 1. Introduction

The most updated available global estimate found approximately 550,000 new bladder cancer (BC) cases being diagnosed every year, making it the 10th most common form of cancer worldwide [1]. The incidence and mortality rates are approximately four times higher among men than women [1]. At the time of diagnosis, the majority of all detected cancers will be confined to the urothelium or lamina propria, whereas a smaller proportion is already muscle-invasive [2]. Radical cystectomy (RC) including pelvic lymphadenectomy remains the standard treatment for muscle-invasive BC (MIBC) patients [3] and for selected NMIBC at highest risk of progression [4]. Approximately 50% of patients with muscle-invasive disease will develop metastases after RC [5]. Despite systemic therapy, metastatic BC confers a high mortality rate with a median survival of 12–15 months and only a 5% 5-year survival rate [6]. In recent years, research has shown the importance of the immune system in cancer treatment. BC is one of the most important cancers that can be successfully treated with the newly emerging immunotherapies [7]. This is particularly important for BC patients who have failed to respond to standard therapy [8]. The advent of the novel immunotherapies was preceded by over 30 years of experience with the first immunotherapy for BC, the successful intravesical therapy with Bacillus Calmette Guerin [9].

In recent years, several markers associated with BC’s biological and clinical behavior have been studied, although most of them are not yet validated [10].

Concerning non-muscle invasive (NMI) BC, the only molecular marker that has been validated is the Bladder Epicheck, a urine test that analyses 15 methylation biomarkers and determines the presence of bladder cancer based on the methylation profile: it has been shown that it could replace urine cytology in the diagnosis of NMIBC [11]. In the context of molecular diagnostics, it also became evident that different cytological categories, classified according to the Paris System for Reporting Urinary Cytology (TPS), also carry a distinct molecular signature [12]. In the follow-up situation of NMIBC patients, cytology and Bladder EpiCheck test in combination may have the potential to reduce cystoscopies in the context of suspicious cytology findings categorized by TPS [13].

Contrary prediction of recurrence, disease progression or survival after RC is primarily based on the Tumor-Node-Metastasis (TNM) classification system [14]. However, TNM is not sufficient to predict individual disease courses and patients within the same TNM stage can have different clinical outcomes following RC [15]. So far, no biomarkers are established for disease prediction after RC in daily clinical practice [5].

There are now several approaches capturing the interaction between neoplasia and the immunological microenvironment to obtain prognostic information in the context of BC. One strategy is the analysis of the immunogenomic landscape, which can be used for prognostic purposes in BC [16]. Significant efforts in cancer immunology have also been directed towards the association of tumor infiltrating lymphocytes (TILs) with disease prognosis in solid cancers [17]. Based on that, Galon et al. developed a classification system called the “IMMUNOSCORE^®^”. This standardized scoring system derived from a measure of CD3 and CD8 cell densities at the tumor center (CT) and invasive margin (IM) [18]. A recently published pooled analysis with over 10,000 patients showed that “IMMUNOSCORE^®^” is significantly associated with the prognosis of patients with colon cancer and also displayed convincing results for gastric and non-small cell lung cancer [19]. In BC, there are only a few studies [20,21,22,23] available but with promising results investigating the prognostic value of TILs in MIBC and the “IMMUNOSCORE^®^” as potential predictor of clinical outcomes after RC. These findings underscore the importance of the host immune system in the clinical outcome of patients with MIBC.

The aim of this study was to modify the known immune score (IS) for analysis of TMA instead of whole tumor slides. Further, we explore the relevance of regulatory T cells (Tregs)-FOXP3—in addition to the established TILs (CD3/CD8 and CD45RO used in IS.

## 2. Materials and Methods

### 2.1. BC Tissue Microarray and Immunohistochemistry

Serial sections of two tissue microarrays (TMAs) containing 318 cystectomy tissue samples from 159 bladder cancer (BC) patients (two tissue cores/patient) were constructed as previously described [24,25]. Pathologists performed the selection of core puncture sites; after inspection of the entire cystectomy specimen, two central areas with histologically confirmed vital tumor were punctured. Specimens were collected between 1990 and 2006 by the Institute of Pathology and Molecular Pathology, University Hospital Zurich, Switzerland. The first TMA consisted of samples from 65 patients and was prepared from a retrospective clinical cohort. The second TMA contained samples from 94 patients and was prepared from a prospective clinical cohort. Both cohorts were approved by the cantonal Ethics Committee (StV-Nr. 25/2008 and 02/2009).

For the current analysis the TMAs were immunohistochemically stained with CD3 (clone: LN10, dilution: 1:200, Leica), CD8 (clone C8/144B, dilution: 1:100, Dako), FOXP3 (clone: FoxP3 236A/E7, dilution: 1:50, Abcam) and CD45RO (clone: UHCL1, dilution: 1:200, Dako) antibodies. The staining was similarly performed as previously described [26]. The stained slides were scanned and imported to QuPath (version 0.1.2, Peter Bankhead, Belfast, Northern Ireland, UK), an open source software for digital pathology image analysis [27]. Automated analysis was performed to detect and quantify each immune cell subpopulation as described in the following steps:

[1] QuPath’s automated “TMA dearrayer” was used to identify tissue cores. The resulting TMA grid was manually verified and amended where necessary.
[2] Stain vector and background estimates were applied to improve stain separation using color deconvolution by QuPath’s “Estimate stain vectors” command.
[3] QuPath’s built-in “Simple tissue detection” and “Fast cell counts” commands were applied. The measurements were visually controlled, and the parameters manually adjusted by a board-certified pathologist (LB) until convincing results could be achieved. In particular the “thresholdDAB value” determining the cut-off for positive cell count had to be adapted in dependence of the different staining intensities of the antibodies.
[4] Output was cumulated, averaged, and reported as positive counts (pc), negative counts (nc), ratio (pc/pc + nc), and density (pc/mm^2^).

### 2.2. Construction of the Modified Immunoscore Prediction Model

The original IS concept consist of a combination of the density and location of CD3 and CD8 positive TIL. For each marker (CD3 and CD8) and each zone (center of tumor and invasive margin) a Score 0–4 is assigned and then cumulated into a summative risk category [28]. To construct our modified Immunoscore (mIS) prediction model, it was necessary to define the scientifically most relevant value from the available source data generated by the above-mentioned immunohistochemistry assays. Based on biological reasoning, the averaged density value of the two tissue cores for CD3, CD8, CD45RO, and FOXP3 per patient was selected to construct the mIS prediction model. Log-transformed average densities were used throughout the analyses to mitigate an undesirable effect of extreme values. Based on that the four parameters were cumulative integrated in the model and the cohort was then dichotomized in high “mIS/favorable risk” and “low mIS/unfavorable risk”.

### 2.3. Statistical Analysis

All statistical analyses were performed using the statistical programming environment R 4.0.3 (R Core Team, Vienna, Austria). Univariable and multivariable Cox regression analysis was used to explore the predictive potential of mIS components (CD3, CD8, CD45RO, FoxP3) with regards to Progression-free survival (PFS) and Cancer Specific Survival (CSS). Follow-up after a cystectomy consisted of annual imaging examinations (first two years normally imaging all 6 month) for five years, after which the follow-up was individualized depending on the age of the patient and their general medical condition.

In a first step, we used the Chi-squared value as a measure to select the most informative combination of mIS components. PFS was defined if local or distant recurrent disease was detected during follow-up. CSS was defined as the time between the date of RC and the date of death from bladder cancer.

In a next step, multivariable analyses were conducted to investigate *(a)* the role of mIS components as independent predictors for the outcomes of interest and *(b)* the performance improvement (concordance index, optimism-corrected by bootstrapping) of the AJCC-only model after addition of mIS components. The American joint committee on cancer classification (AJCC) staging systems represents the most widely used and accepted method [14]: like the International Union against Cancer-(UICC) System, it is based on the Tumor size, Lymph Nodes affected, and Metastases (TNM)-scoring system. Both the European Association of Urology (EAU) and the American Association of Urology (AUA) recommend this system for the staging of bladder cancer [29,30]. The AJCC-stating system consists of eight stages (0a–IVB). Therapeutic decisions and prognosis are guided and influenced by this classification. Patients treated with neoadjuvant chemotherapy were scored according to the TNM-system into the highest stage resulting from the most recent transurethral resection before neoadjuvant therapy or based on the histology of the cystectomy specimen, whichever was higher.

As a last step and described above we divided the whole cohort into “high mIS/favorable risk” and “low mIS/unfavorable risk” based on our multivariable model and evaluated the potential of mIS to sub-stratify patients within AJCC stages by Kaplan–Meier analysis. Due to the limited number of patients with non-muscle invasive disease (0a, 0is, and I) who underwent RC (Those were either BCG-non-responders or were at highest risk of progression and upfront cystectomy as an option was discussed and performed) we chose to group them together into a pooled stage (AJCC 0a/0is/I). The log-rank test was used to compare the survival curves. P values lower than 0.05 were considered statistically significant (two-sided). A sufficiently powered internal validation by a split-sample approach was not feasible (see Supplemental Files).

## 3. Results

### 3.1. Patient Characteristics

A total of 158 patients were included in this study after exclusion of one patient with missing information on nodal staging (Table 1). The cohort consisted of 122 (77.2%) male and 36 (22.8%) female patients. The median age at diagnosis was 68 years (range 44–87). A total of 31 patients (19.6%) were followed for AJCC stage 0a/0is/I tumor, 34 patients (21.5%) for a stage II tumor, 57 patients (36.1%) for IIIa and 36 patients (22.8%) for IIIb stage tumor. In 68 patients (43.1%) concomitant CIS was detected. High grade disease was present in almost all patients (n = 149, 94.3%). Lymph node metastases were present in 53 (33.6%) patients. Neoadjuvant chemotherapy was performed in seven patients (4.4%). Median follow-up was 36 months (range: 0.1–111.3).

### 3.2. Immunhistochemistry

**Figure 1:**
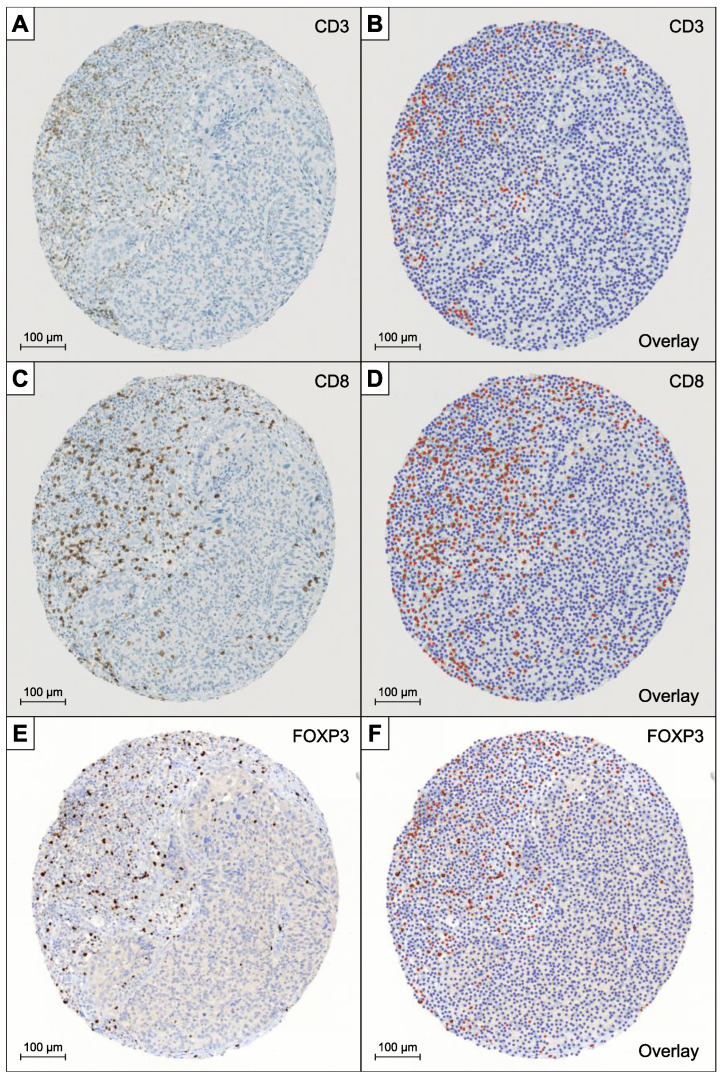
Overview of exemplary samples of scanned TMA-sections stained with antibodies for CD3 (**A**), CD8 (**C**), and FOXP3 (**E**) and the corresponding overlays generated by QuPath (**B**,**D**,**F**). Red dots highlight the lymphocytes that where rated positive, while the blue dots are showing the remaining detected cells that were counted as negative.

Figure 1 shows representative samples of scanned TMA-sections stained with antibodies for CD3 (A), CD8 (C), and FOXP3 (E) and the corresponding overlays generated by QuPath (B, D, and F). The good staining quality allowed an automated evaluation of all patients without any further changes after adapting the basic parameters for “Simple tissue detection” and “Fast cell counts” for the whole section as well as setting the “thresholdDAB value” for each immunohistochemical staining.

**Figure 2:**
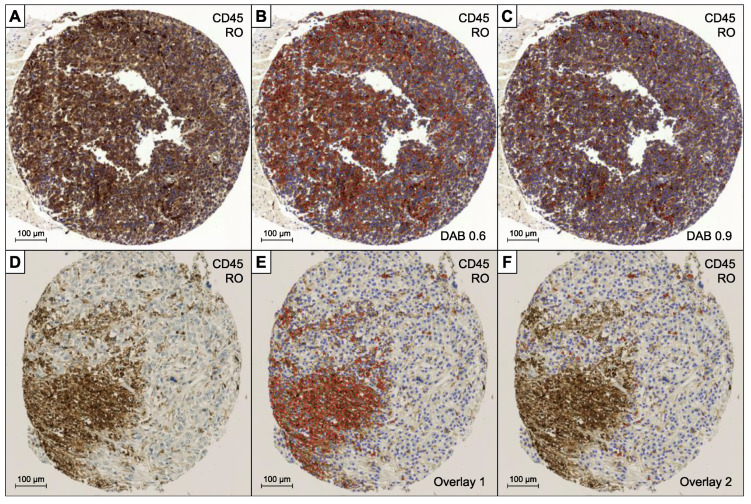
Staining of CD45RO: (**A**) Strong cytoplasmic staining in tumor cells. (**B**) Automated evaluation “overlay”. (**C**) Manually altered threshold for the automated evaluation. (**D**) Diffuse staining of necrotic area. (**E**) Automated evaluation “overlay”. (**F**) Manually altered staining area.

The sections stained with CD45RO (an example is depicted in Figure 2) were more difficult to evaluate due to artificial cytoplasmic staining of tumor cells in some cases and diffuse unspecific staining in necrotic areas. An example with quite strong cytoplasmic staining in tumor cells is shown (A), in which the evaluation with the usual parameters would have resulted in a positive cell count much too high (B). The “thresholdDAB value” had to be increased until the tumor cells were rated as negative and only the very dark colored lymphocytes were counted as positive (C). Another example is depicted (D) with a large necrotic area showing diffuse staining that was counted positive (E). The area had to be manually deleted for a reliable evaluation (F).

### 3.3. Univariable Analysis

A univariable analysis to evaluate the prognostic potential of CD3, CD8, CD45RO, and FOXP3 cell densities in the TMA cores in order detect the most informative predictors and the optimal form of incorporation in the mIS model was performed. Among the mIS parameters, all showed a significant association with PFS and CSS in univariable analysis (Table 2) with the exception for CD3 that only showed a significant association with CSS but not with PFS. Furthermore, higher AJCC stages and known pathological features as perineural infiltration (Pn), vascular infiltration (V), and presence of nodal disease (L) showed a significant shorter PFS and CSS in univariable analysis. Concurrent carcinoma in situ (CIS), age and sex of the patient were not associated with PFS and CSS. Finally, we performed univariable cox regression analysis within the overall cohort stratified by high mIS versus low-mIS and plotted Kaplan–Meier curves for PFS and CSS (Supplemental Figures S1 and S2). Statistically significant better survival data for the high mIS group were observed. However, it should be noted that the results have to be interpreted with caution since mIS was fitted to the same cohort.

### 3.4. Multivariable Analysis

In a multivariable Cox proportional hazards model, incorporating AJCC Stages and one mIS component (average densities for CD3, CD8, CD45RO and FOXP3), mIS components remained a significant predictor for a longer PFS and CSS, with the exception of CD45RO that was only a significant predictor for PFS. By incorporating three or all mIS components in the model only FOXP3 remained a significant predictor for longer PFS and CSS (Table 3). Further analyses revealed that addition of mIS components considerably increases the concordance index of the AJCC-only model, even after correcting for overfitting, indicating improved predictive potential of the full model (see Supplemental Files).

### 3.5. AJCC Sub-Stratification

**Figure 3:**
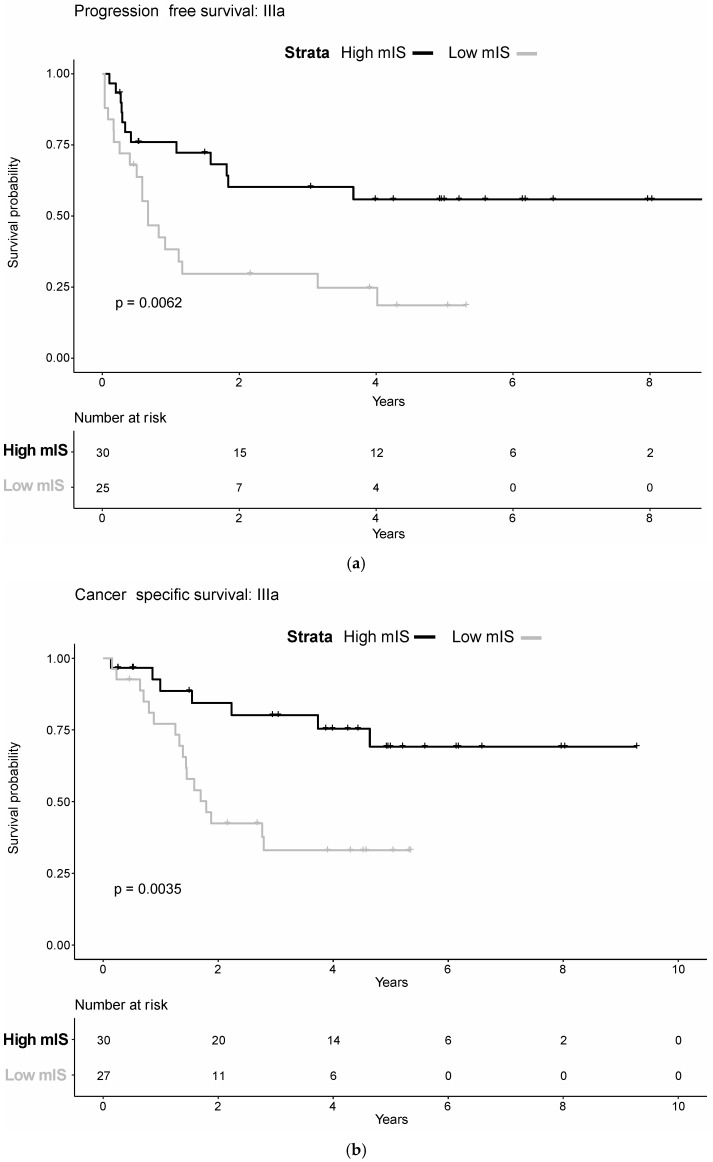
(**a**). Kaplan–Meier curve of progression-free survival for high mIS (black line) and low mIS (grey line) in AJCC-stage IIIa. (**b**). Kaplan–Meier curve of cancer-specific survival for high mIS (black line) and low mIS (grey line) groups in AJCC-stage IIIa.

Next, we tested if mIS could predict disease course if patients were substratified in different AJCC stages. Therefore, we applied the mIS (high mIS vs. low mIS) among the different AJCC stages and tested for prediction for PFS and CSS. Patients in stage IIIa with high mIS showed a significantly longer PFS (Hazard Ratio (HR): 2.7; *p* = 0.008) and CSS (HR: 3.5; *p* = 0.006) as compared to patients with low mIS. In contrast, PFS and CSS did not differ significantly between high and low risk mIS within other AJCC stages (Figure 3a,b and Supplemental Figures S4a,b, S5a,b and S6a,b).

## 4. Discussion

The present study shows the independent prognostic significance of mIS components in a representative cohort of patients with BC undergoing RC. Furthermore, the mIS showed to be of additional value for the AJCC-TNM classification as mIS was able to stratify patients into low- and high-risk groups for PFS and CSS within the AJCC stage IIIa. Additionally, we could demonstrate that all of the mIS components showed a significant association with PFS and CSS with the exception for CD3 that showed only a significant association with CSS. In the multivariable analysis after incorporating three or all mIS components, we see that only FOXP3 remains an independent significant predictor for longer PFS and CSS. We also noted that mIS was not able to stratify patients further within the AJCC stage IIIb, as most likely the presence of nodal disease is the main prognostic marker within this group. Our cohort can be considered representative, as the AJCC staging classification system and known worse pathological features (Pn infiltration, V infiltration, and N Status) were associated with shorter survival outcomes in univariable analysis.

Previous work has shown that TIL play a role as prognostic marker in BC patients after RC. Most of these studies are in line with the findings of the present investigation. Sharma et al. [21] analyzed the link between TIL and BC prognosis. In their subanalysis of 31 patients with muscle-invasive disease, they found a significant association between high central tumor TIL CD8 density and survival in MIBC. In contrast to our methodology, they used whole slide tumor specimens for their analysis and focused on central-tumor infiltration. This is in contrast to our work, where we used TMAs and could therefore not further differentiate between central-tumor and peri-tumoral stromal infiltration. Ingels et al. [22] performed a similar study on transurethral resection specimens in a cohort of 10 patients with pT1 and 20 patients with MIBC and analyzed peri-tumoral stromal infiltration of CD3 and CD8 cells. Their survival analysis showed a significantly better survival among CD3- and CD8-infiltrated tumors. Despite our different approach using TMAs instead of whole tumor slides our results confirm the prognostic role of TIL in RC patients and suggest that the prognostic value of TIL is independent from localization within or around the tumor.

To our knowledge, this is the second study where the association of Tregs, T effector and T memory cells with survival outcome in BC patient after RC was analyzed using a TMA approach.

Horn et al. [23] investigated in a cohort of 149 cystectomy patients with a median follow-up of 46 months whether the infiltration densities of a selected set of adaptive immune markers on TMAs were associated with certain clinicopathological parameters. In their study, it was demonstrated that high ratios of FOXP3+/CD3+ lymphocytes and FOXP3+/CD8+ lymphocytes were significantly associated with inferior survival outcome As opposed to our findings, the infiltration densities of individual markers (CD3, CD8, FOXP3, and CD45RO) were not significantly associated with survival outcome but high CD3 and CD8 infiltration showed trends towards better prognosis. In our dataset densities of all the above-mentioned markers were significantly associated with CSS; CD8, FOXP3, and CD45RO with PFS in the univariable analysis.

When we evaluated FOXP3 individually as a prognostic marker it is interesting to note that higher infiltration densities of FOXP3 was among the strongest predictors for favorable PFS and CSS. FOXP3 is known as one of the best singular marker for Tregs [31]. In tumor immunity, Tregs are involved in tumor development and progression by inhibiting antitumor immunity, therefor a high infiltration by Tregs is often associated with poor survival in various types of cancer [32]. Conflicting results have been reported, Horn et al. showed unfavorable survival outcomes associated with higher ratios of FOXP3/CD3 and FOXP3/CD8 infiltration densities. However, the observation made by Salama et al. [33] that displays better survival outcome for colorectal cancer patients with a high density of FOXP3 is therefore counter-intuitive and contrasts with what has been reported for other solid tumor types, including melanoma, breast, ovarian, hepatocellular, and pancreatic cancers. Winerdal et al. [34] found that a higher density of FOXP3 expressing TIL is correlated with better OS and PFS in a cohort of RC patients. A possible explanation offered by the authors was that these TIL are not true Tregs but rather activated T-cells, with up-regulated FOXP3 expression. In our study we also observed that higher infiltration densities of FOXP3 was among the strongest predictors for favorable PFS and CSS, therefore our results support the alternative significance of FOXP3 expressing TIL in different solid tumors and specifically in BC.

Regarding the role of CD45RO, Mlecnik et al. [35] showed that a high density of this T cell subpopulation was correlated with longer survival rates in colorectal cancer patients. These results support our findings of longer PFS and CSS in BC patients with higher CD45RO densities in the univariate analysis.

Our findings highlight the importance of the adaptive immune response in cancer control of the host as long the tumor is locally confined, correlation of TIL densities and outcome are probably no longer linked in nodal disease (Stage IIIb).

The present study has some limitations. Based on the retrospective nature of our study data analysis is susceptible to selection bias. Second, the use of tissue microarrays, the determination of TIL density in two 1mm diameter cores instead of whole tumor slides could be a potential bias, due to the heterogeneity of the tumor and the uneven distribution of TIL. We have to be aware that the TMA cores only represent a small proportion of the whole tumor. Nevertheless, the prognostic significance of TIL found on the TMAs highlights the aspect that determination of mIS in TMAs is feasible and applicable. Third, our cohort is characterized of a low number of patients that received neoadjuvant chemotherapy prior RC. Therefore, no statement about the association between mIS and neoadjuvant chemotherapy can be made.

In conclusion we were able to show that quantitative immunologic signatures for MIBC are of prognostic relevance for survival outcome after radical surgery. Specifically, we could demonstrate that it was possible to stratify patients into low- and high-risk groups within the AJCC stage IIIa, this could be of major importance in selecting patients for emerging adjuvant therapeutic strategies in the treatment of MIBC.

## Figures and Tables

**Table 1:** Patient and tumor characteristics.

Variable	Categorization	n Analyzable	Percent
Total (n = 158) ^†^			
Age at diagnosis Median Follow-up	Median, Range 68 (44–87) 36 months (Range 0.1–111.3)		
Sex	Male	122	77.2
	Female	36	22.8
Tumor Stage	pTa	8	5.1
	pTis	1	0.6
	pT1	26	16.5
	pT2	40	25.3
	pT3	55	34.8
	pT4	28	17.7
Histological Grade ^‡^	missing	2	1.3
	G2	7	4.4
	G3	149	94.3
Histological Grade ^§^	missing	2	1.3
	Low Grade	7	4.5
	High Grade	149	94.3
Adjacent Carcinoma in situ	Yes	68	43.1
	No	90	57.0
Perineural Invasion	missing	66	41.8
	Yes	23	14.6
	No	69	43.7
Vascular Invasion	Yes	32	20.3
	No	126	79.7
Lymph-vascular Invasion	Yes	56	35.4
	No	102	64.6
Lymph Node metastasis	Yes	53	33.6
	No	105	66.5
AJCC Stage	0a	8	5.1
	0is	1	0.6
	I	22	13.9
	II	34	21.5
	IIIa	57	36.1
	IIIb	36	22.8
Neoadjuvant Chemotherapy	Yes	7	4.4
	No	151	95.6

^†^ After exclusion of one patient with missing information on nodal staging. ^‡^ Staging and grading according to the 1973 WHO classification system. ^§^ Staging and grading according to the 2004 WHO classification system. AJCC = American Joint Committee on Cancer.

**Table 2:** Univariable Cox-Regression Analysis.

Variable	PFS:HR	95% CI	*p* Value	CSS:HR	95% CI	*p* Value
*Clinical*						
Age (continuous per year)	1.00	0.98–1.03	0.7669	1.00	0.97–1.03	0.9467
Gender						
Female	*ref*			*ref*		
Male	1.16	0.69–1.95	0.5805	1.17	0.65–2.11	0.5909
*Pathological*						
Grade ^†^ (continuous G2/G3)	1.23	0.39–3.91	0.7266	0.99	0.31–3.18	0.9929
Cis	0.99	0.62–1.5	0.546	1.21	0.73–2.02	0.4616
Pn	4.61	2.39–8.88	**<0.0001**	4.58	2.27–9.21	**<0.0001**
V	2.42	1.41–4.13	**0.0013**	2.71	1.55–4.74	**0.0005**
LVI	3.50	2.19–5.58	**<0.0001**	4.65	2.72–7.92	**<0.0001**
*AJCC-Stages*						
0a/0is/I	*ref*			*ref*		
II	1.59	0.64–3.95	0.3194	0.87	0.28–2.74	0.8092
IIIa	3.26	1.50–7.11	**0.0029**	2.60	1.12–6.05	**0.0263**
IIIb	6.69	2.96–15.14	**<0.0001**	7.51	3.18–17.76	**<0.0001**
*Immuno-histological markers*						
CD3 ^‡^ (continuous)	0.89	0.79–1.01	0.690	0.82	0.72–0.95	**0.0060**
CD8 ^‡^ (continuous)	0.82	0.72–0.94	**0.0054**	0.75	0.64–0.88	**0.005**
CD45RO ^‡^ (continuous)	0.78	0.65–0.93	**0.0063**	0.77	0.63–0.93	**0.0082**
FOXP3 ^‡^ (continuous)	0.74	0.60–0.91	**0.0036**	0.65	0.52–0.81	**0.0001**
*Modfied Immunoscore (mIS)*						
Low mIS	*ref*					
High mIS	0.43	0.27–0.70	**0.0005**	0.33	0.18–0.57	**<0.0001**

^†^ Grading according to the 1973 WHO classification system. ^‡^ Log-transformed and truncated. AJCC = American Joint Committee on Cancer/PFS:HR = Progression Free Survival Hazard Ratio/CSS:HR = Cancer Specific Survival Hazard Ratio/Pn = Perineural Invasion/V = Vascular Invasion/LVI = Lymph Vascular Invasion.

**Table 3:** Multivariable Cox-Regression Analysis/AJCC model + all mIS components.

Variable	PFS.HR	95% CI	*p* Value	CSS.HR	95% CI	*p* Value
*AJCC-Stages*						
0a/0is/I	*ref*			*ref*		
II	2.24	0.87–5.76	0.0947	1.29	0.39–4.23	0.6754
IIIa	3.93	1.77–8.71	**0.0008**	3.21	1.36–7.58	**0.0079**
IIIb	7.53	3.24–17.5	**<0.0001**	9.30	3.77–22.92	**<0.0001**
*Immuno-histological markers*						
CD3	1.07	0.86–1.35	0.5422	0.91	0.71–1.16	0.4373
CD8	1.03	0.82–1.28	0.8262	0.94	0.74–1.18	0.5732
CD45RO	0.86	0.60–1.23	0.3957	1.23	0.83–1.83	0.2926
FOXP3	0.70	0.52–0.95	**0.0208**	0.64	0.46–0.89	**0.0080**

AJCC = American Joint Committee on Cancer/PFS:HR = Progression Free Survival Hazard Ration/CSS:HR = Cancer Specific Survival Hazard Ratio/Pn = Perineural Invasion/V = Vascular Invasion/LVI = Lymph Vascular Invasion.

## Data Availability

The data presented in this study are available on request from the corresponding author.

## Supplementary Materials

The following supporting information can be downloaded at: https://www.mdpi.com/article/10.3390/diagnostics12061360/s1, Supplemental files: Overview of Statistical Analysis. Figure S1: Progression-free survival: all patients, Figure S2: Cancer-specific survival: all patients, Figure S3: Progression-free survival and Cancer-specific survival: 0a/0is/I, Figure S4: Progression-free survival and Cancer-specific survival: II, Figure S5: Progression-free survival and Cancer-specific survival: IIIb.
