# Supplementary material for: Modified Immunoscore Improves Prediction of Survival Outcomes in Patients Undergoing Radical Cystectomy for Bladder Cancer—A Retrospective Digital Pathology Study
Source: Diagnostics (Basel). 2022 Jun 1;12(6):1360. doi: 10.3390/diagnostics12061360 (PMC9222135; doi:10.3390/diagnostics12061360)
Supplement: Supplementary file 1 [file diagnostics-12-01360-s001.zip › Supplemental figures.pdf]

*Diagnostics*

supplemental figures

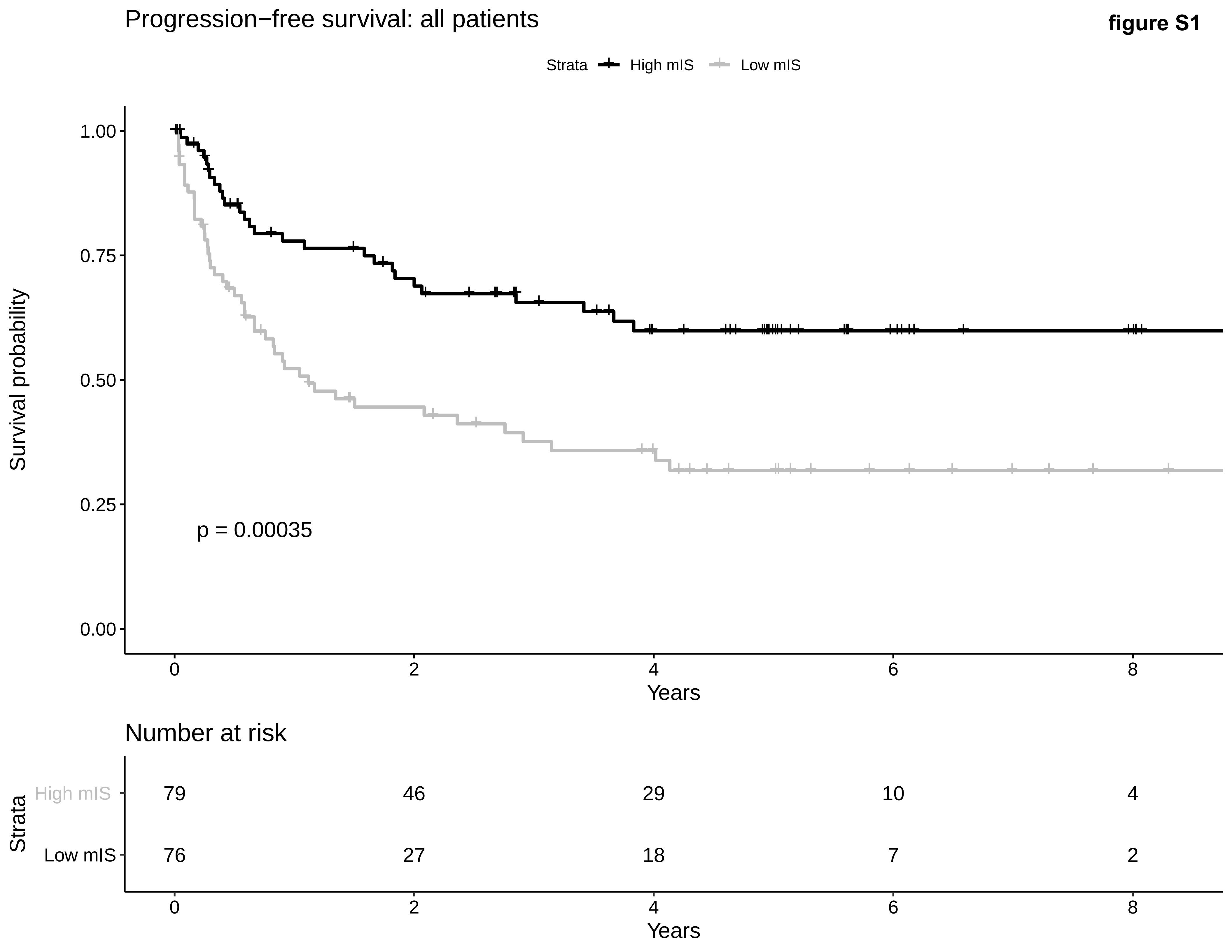

Progression−free survival: all patients
figure S1
Strata
High mIS
Low mIS
Survival probability
1.00
0.75
0.50
0.25
0.00
p = 0.00035
0
2
4
6
8
Years
Number at risk
Strata
High mIS
79
46
29
10
4
Low mIS
76
27
18
7
2
0
2
4
6
8
Years

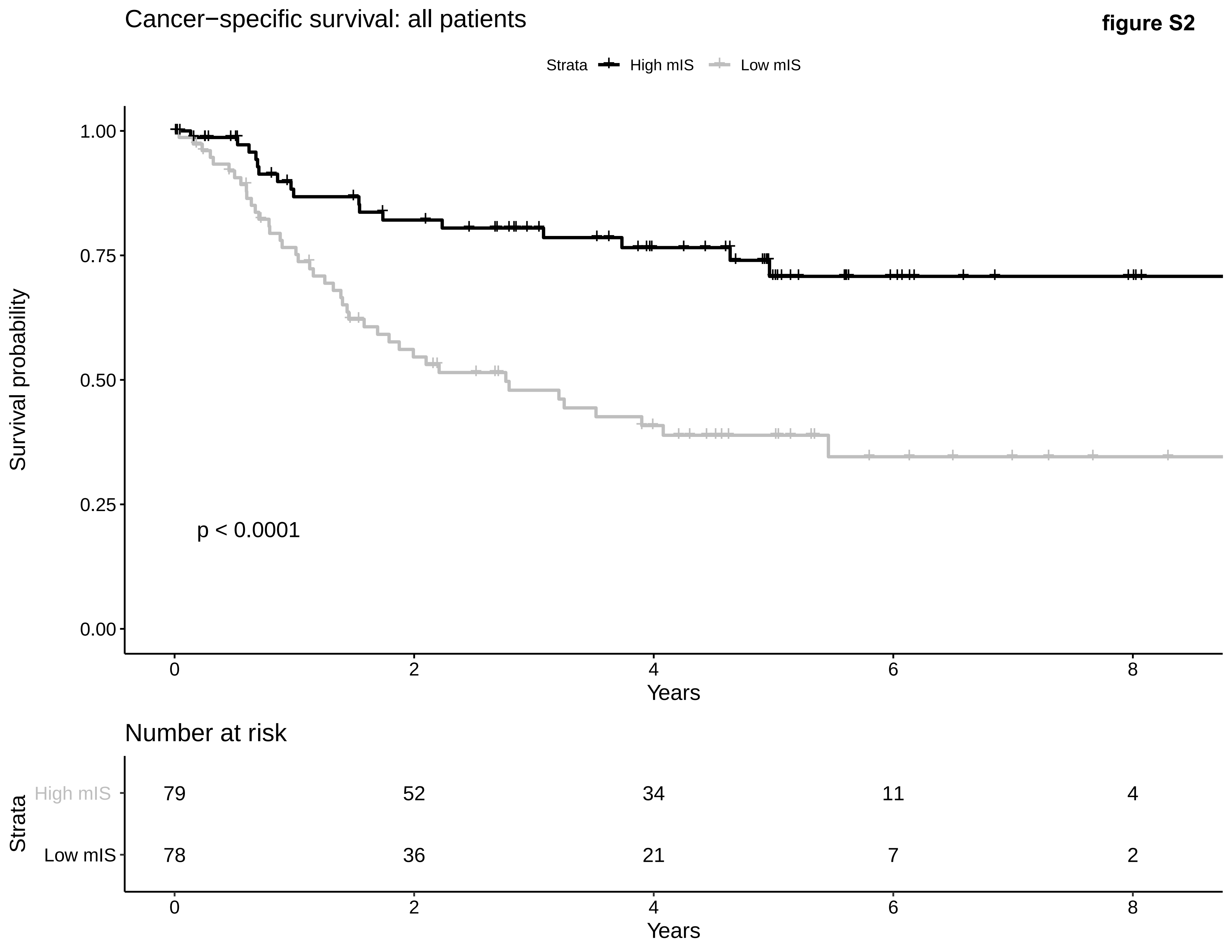

Cancer−specific survival: all patients
figure S2
Strata
High mIS
Low mIS
Survival probability
1.00
0.75
0.50
0.25
0.00
p < 0.0001
0
2
4
6
8
Years
Number at risk
Strata
High mIS
79
52
34
11
4
Low mIS
78
36
21
7
2
0
2
4
6
8
Years

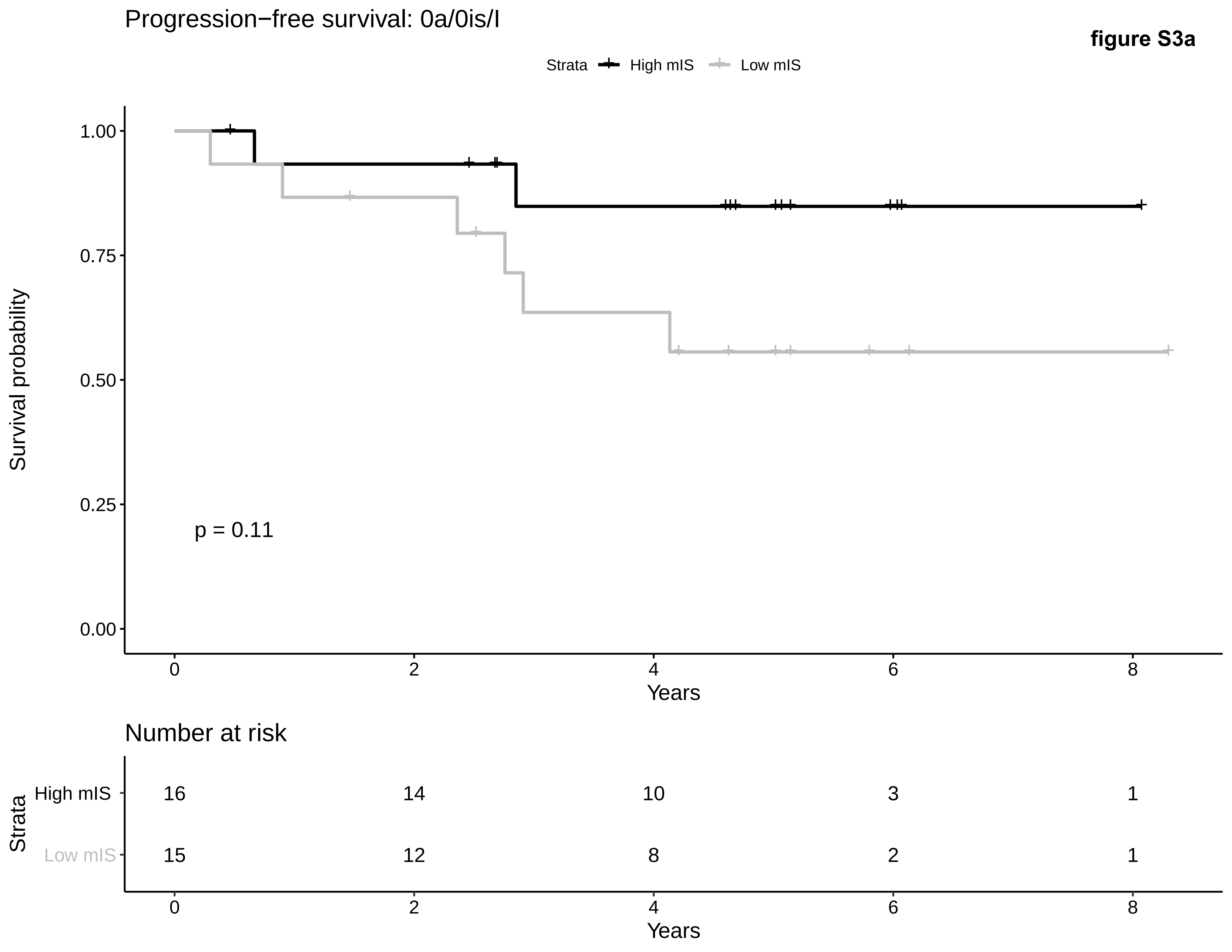

Progression−free survival: 0a/0is/I
figure S3a
Strata
High mIS
Low mIS
1.00
0.75
0.50
0.25
0.00
Survival probability
p = 0.11
0
2
4
6
8
Years
Number at risk
Strata
High mIS
16
14
10
3
1
Low mIS
15
12
8
2
1
0
2
4
6
8
Years

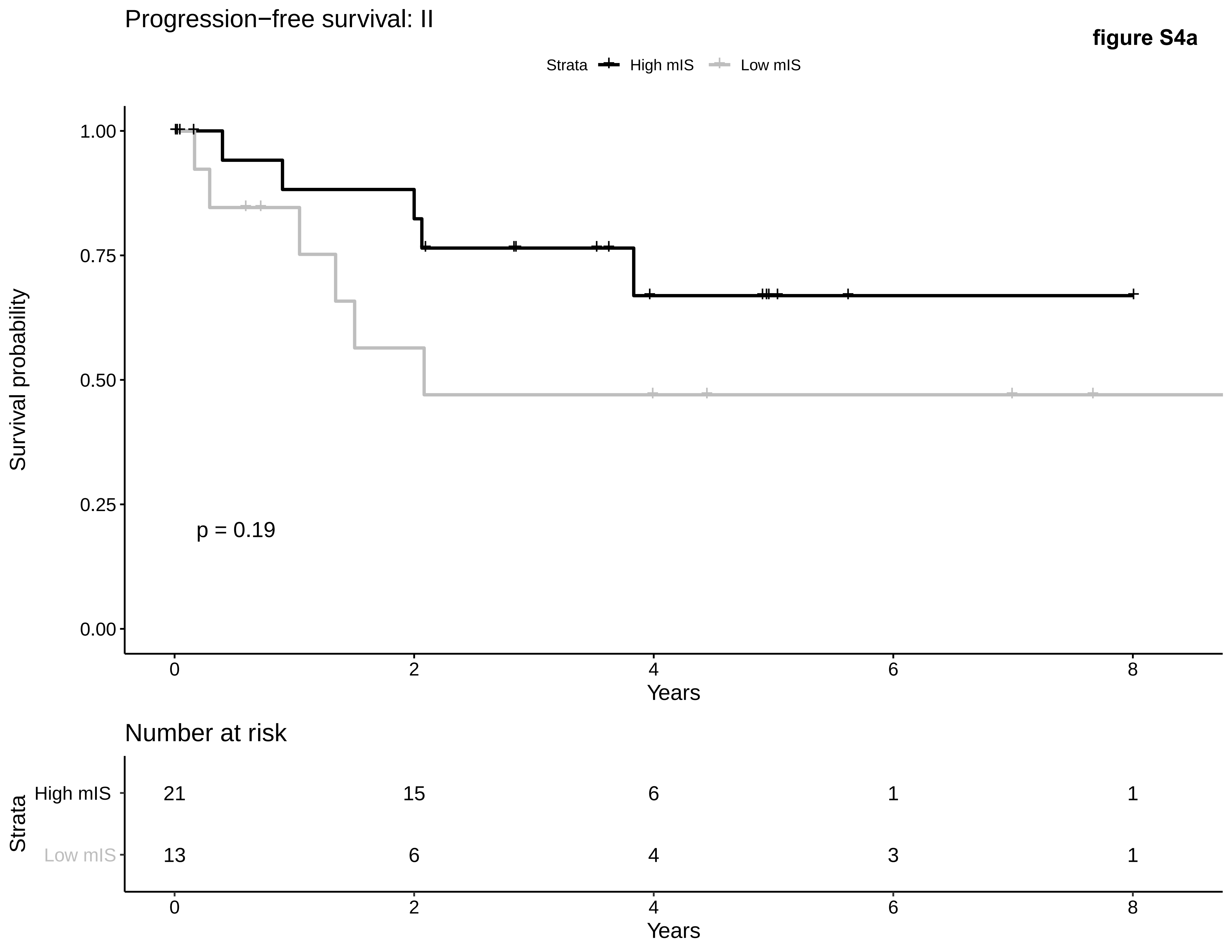

Progression−free survival: II
Strata
High mIS
Low mIS
Survival probability
1.00
0.75
0.50
0.25
0.00
p = 0.19
0
2
4
6
8
Years
Number at risk
Strata
High mIS 21 15 6 1 1
Low mIS 13 6 4 3 1
0
2
4
6
8
Years

**figure S5a**

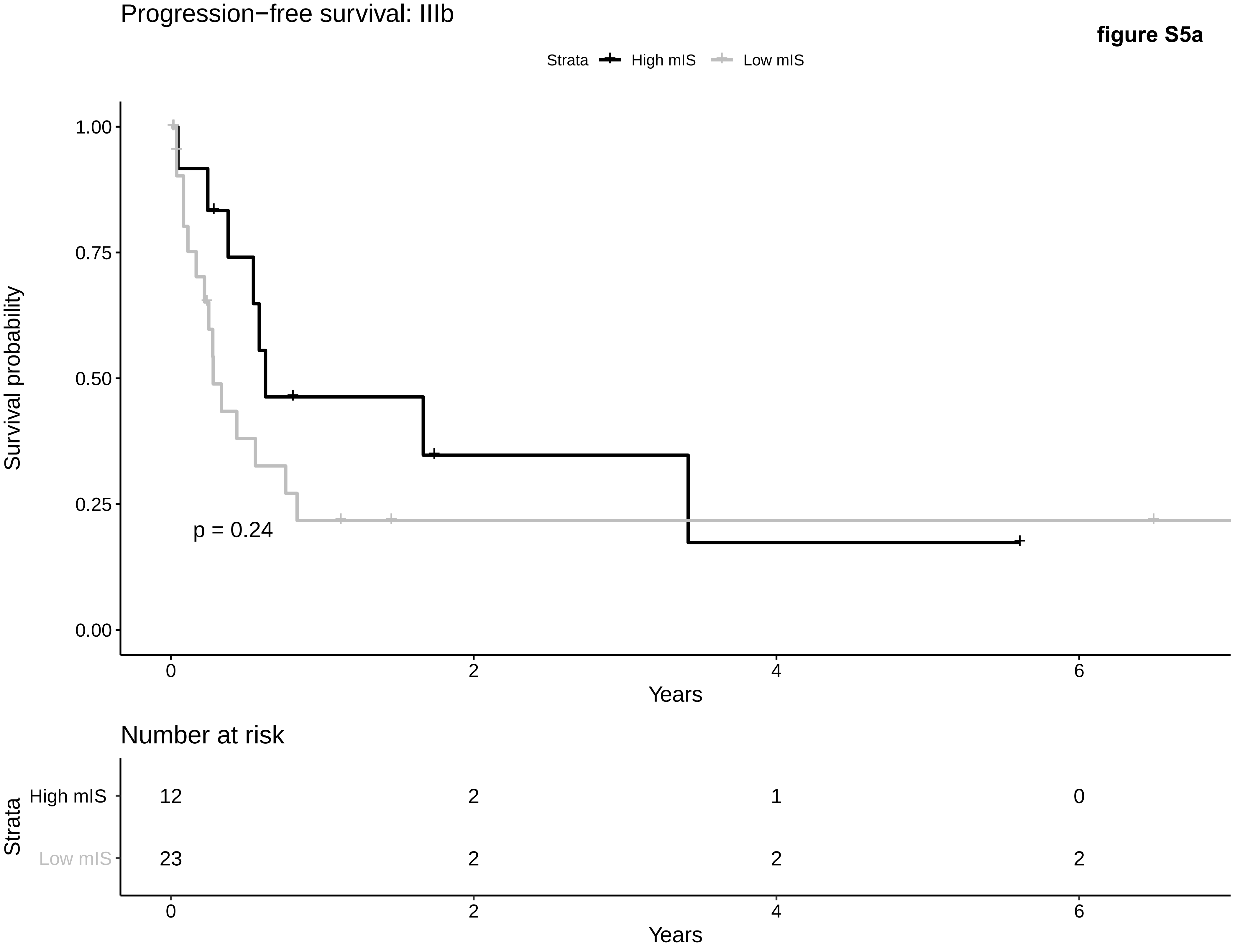
Progression−free survival: IIIb
Strata
High mIS
Low mIS
1.00
0.75
0.50
0.25
0.00
Survival probability
p = 0.24
0
2
4
6
Years
Number at risk
Strata
High mIS
Low mIS
12
2
1
0
23
2
2
2
0
2
4
6
Years

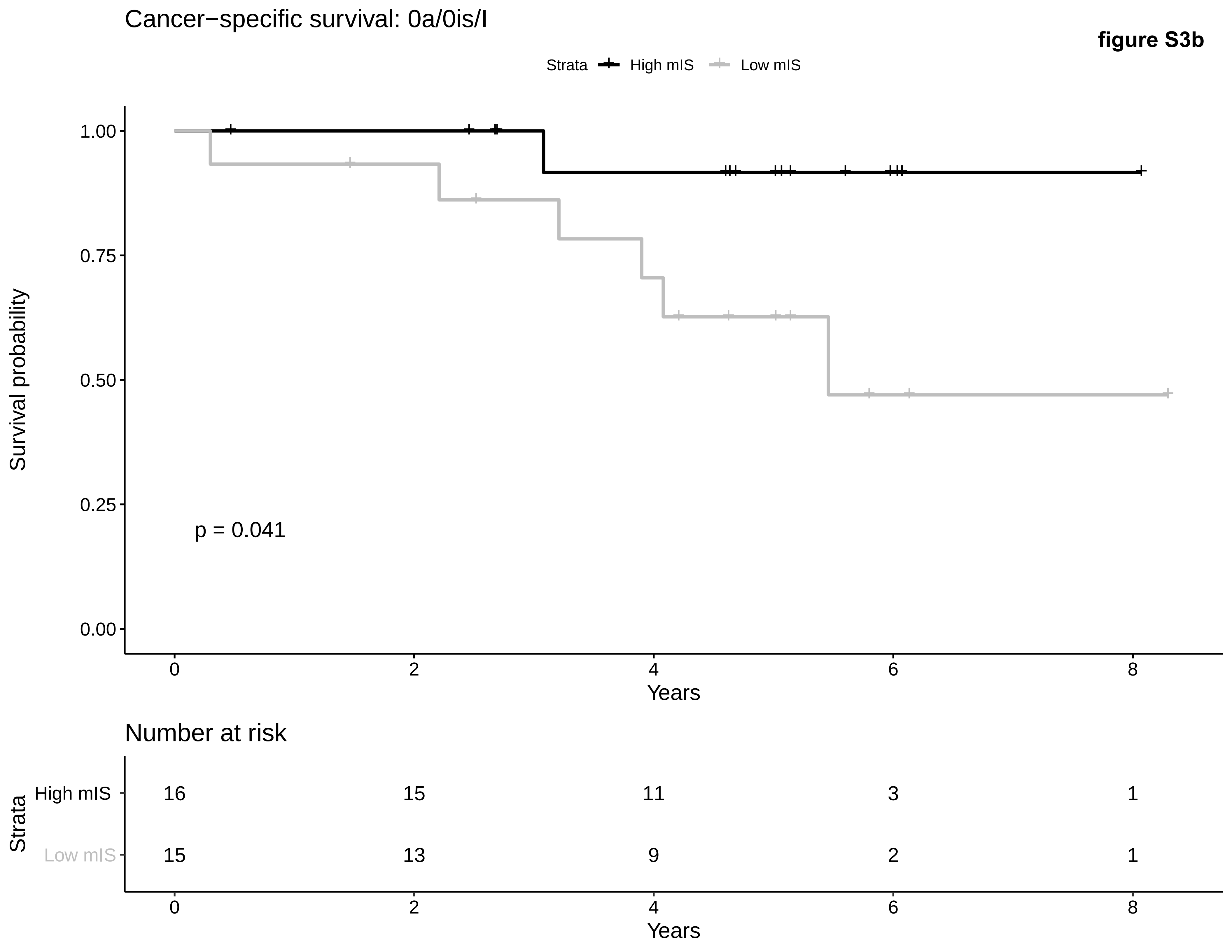
Cancer−specific survival: 0a/0is/I
figure S3b
Strata
High mIS
Low mIS
Survival probability
1.00
0.75
0.50
0.25
0.00
p = 0.041
0
2
4
6
8
Years
Number at risk
Strata
High mIS
Low mIS
16
15
11
3
1
15
13
9
2
1
0
2
4
6
8
Years

**figure S4b**

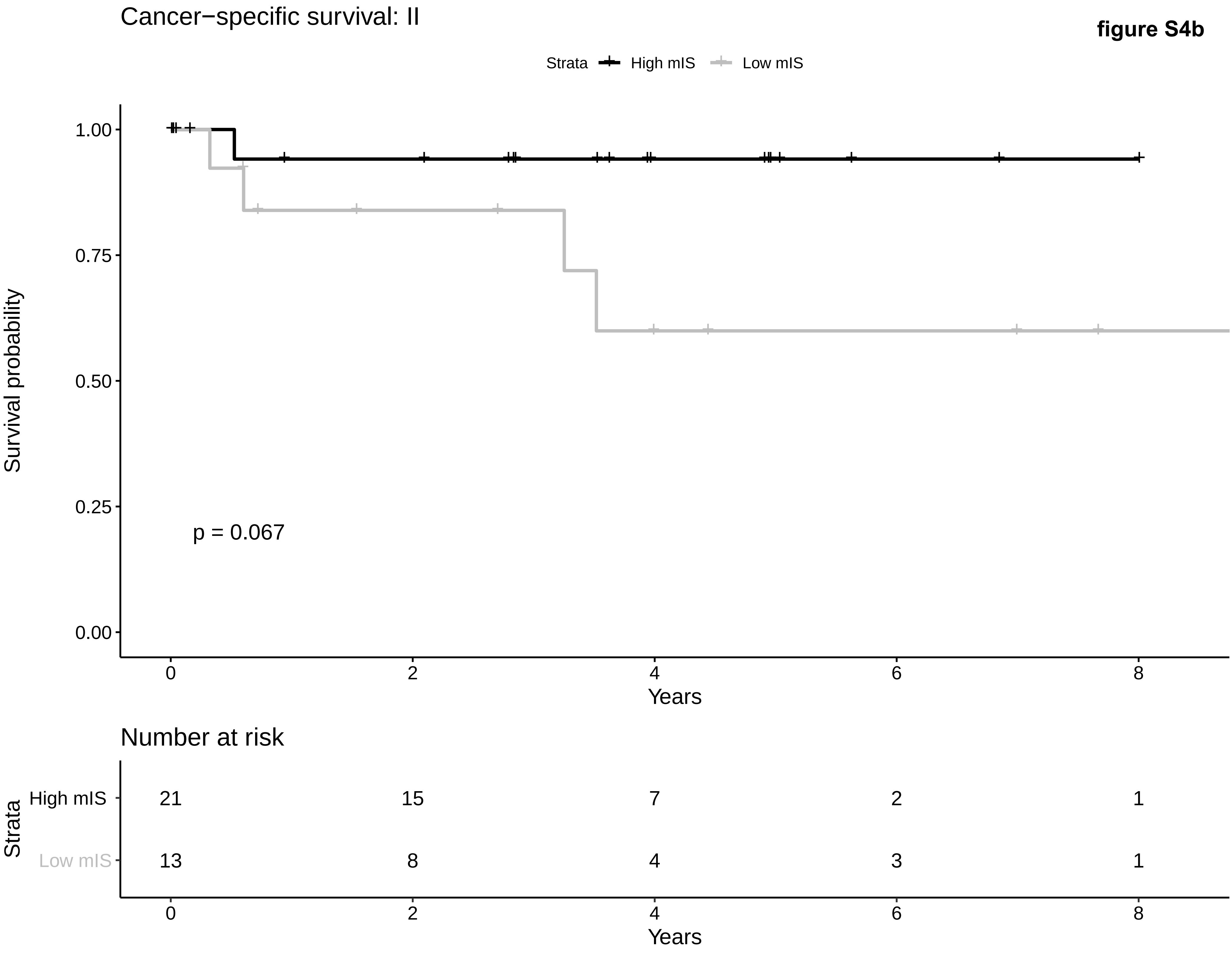
Cancer−specific survival: II
Strata
High mIS
Low mIS
Survival probability
1.00
0.75
0.50
0.25
0.00
p = 0.067
0
2
4
6
8
Years
Number at risk
Strata
High mIS
Low mIS
21
15
7
2
1
13
8
4
3
1
0
2
4
6
8
Years

**figure S5b**

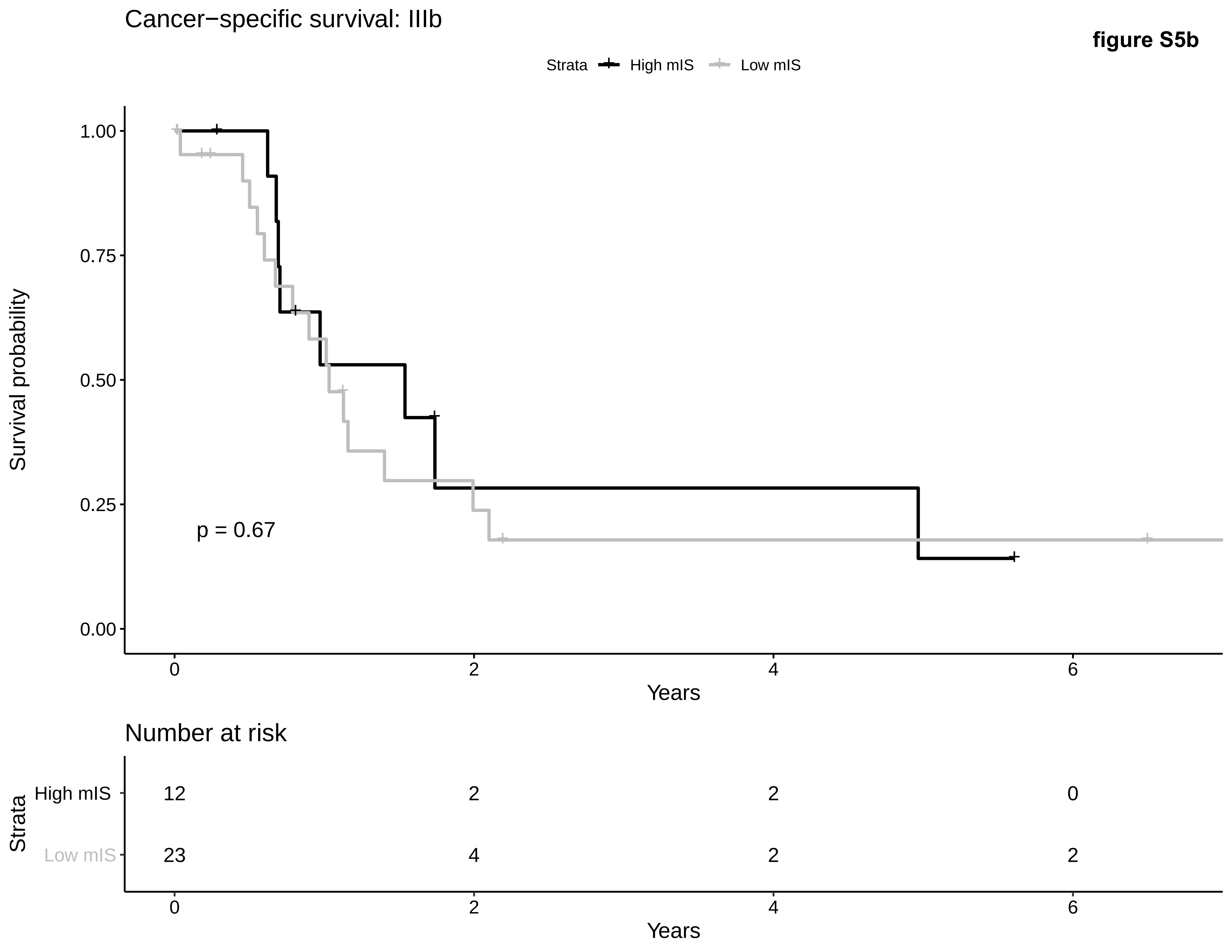

Cancer−specific survival: IIIb
Strata
High mIS
Low mIS
1.00
0.75
0.50
0.25
0.00
Survival probability
p = 0.67
0
2
4
6
Years
Number at risk
Strata
High mIS
Low mIS
12
2
2
0
23
4
2
2
0
2
4
6
Years
