# Supplementary material for: Modified Immunoscore Improves Prediction of Survival Outcomes in Patients Undergoing Radical Cystectomy for Bladder Cancer—A Retrospective Digital Pathology Study
Source: Diagnostics (Basel). 2022 Jun 1;12(6):1360. doi: 10.3390/diagnostics12061360 (PMC9222135; doi:10.3390/diagnostics12061360)
Supplement: Supplementary file 1 [file diagnostics-12-01360-s001.zip › Supplemental files.pdf]

Diagnostics

Modified Immunoscore improves prediction of survival outcomes in patients undergoing radical cystectomy for bladder cancer. A retrospective digital pathology study

Uwe Bieri (1), Lorenz Buser (2), Marian S. Wettstein (1), Daniel Eberli (1), Karim Saba (1), Holger Moch (2), Thomas Hermanns (1), Cédric Poyet (1)*

(1) Department of Urology, University Hospital of Zurich, University of Zurich, Zurich, Switzerland
(2) Institute of Surgical Pathology, University Hospital of Zurich, University of Zurich, Zurich, Switzerland

*Corresponding author:
Email: Cedric.Poyet@usz.ch

Overview of statistical Analysis

Ver. 2.0

Exploration of dataset (completeness and missingness)

Exlcusion of 4 cases flagged as “rule out”.
Available observations: 159

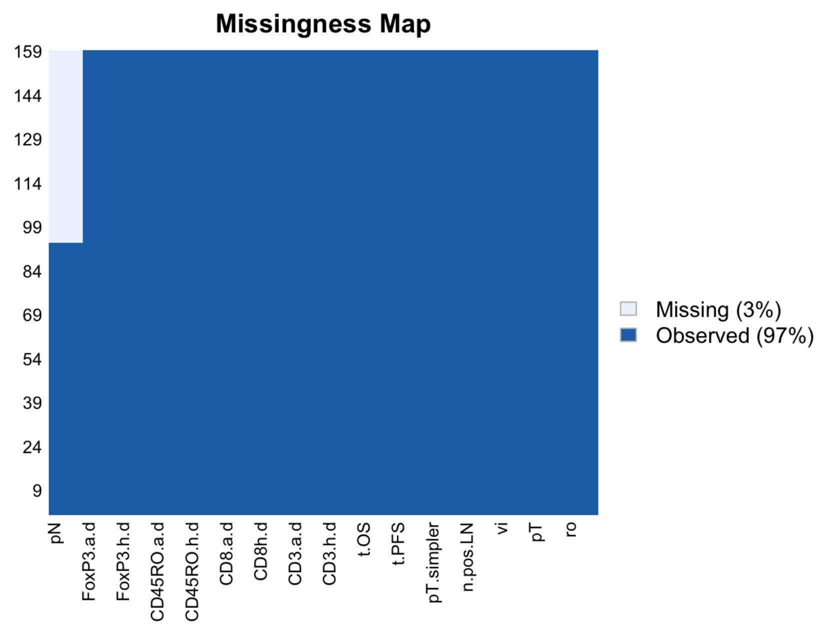

Missingness map (renamed variables): pN and pne manifest a certain missingess

Exploration of event rates to determine power of sample

Progression-free survival (PFS), number of events: 74
Cancer-specific survival (CSS), number of events: 60
Overall survival (OS), number of events: 89

Methdodology

Research question

Among patients undergoing radical cystectomy, can the immunoscore (CD3, CD8, CD45RO, FoxP3) add additional predictive information on top of the already established AJCC staging system?

Modeling approach

- Using the N/10 rule, we can fit between 6 and 9 predictors to the planned Cox proportional hazards model.
- We can only spend 1 to 2 non-immunoscore predictors to fully explore the predictive potential of the immunoscore.
- Non-immunoscore predictors will be summarized into AJCC stage groups.
- Exploration of immunoscore:
  - Current dataset has a high potential to end up in a fishing mission which might be highly damaging.
  - Modeling should be very conservative and knowledge-driven.
  - Value with highest validity from a biological perspective: Density of average value
  - Normalization and truncation if required
  - Evaluation of correlation matrix to screen for collinearity
  - Univariable analysis (driven by chi-squared statistic) to detect the most informative predictors and the optimal form of incorporation
  - Complete univariable analysis to evaluate effect size in relation to other known predictors
  - Multivariable analysis:
    - Role of immunoscore components as independent predictors
    - Evaluation of performance improvement (concordance index) of AJCC-only model and after addition of immunoscore components + Potential of immunoscore to substratify AJCC stages into “high immunoscore / favourable risk” and “low immunoscore / unfavourable risk”.

Derivation of AJCC stages

```
##
##   0a  0is    I   II IIIa IIIb
##    8    1   22   34   57   36
```

(After exlcusion of one patient with missing information on nodal staging.)

Stages 0a and 0is only have 9 observations in total. Therefore they will be collapsed with stage I into a stage 0a/0is/I (AJCCsimplified).

```
##
## 0a/0is/I       II     IIIa     IIIb
##       31       34       57       36
```

Exploration of survival stratified by AJCC stages

Full AJCC

PFS

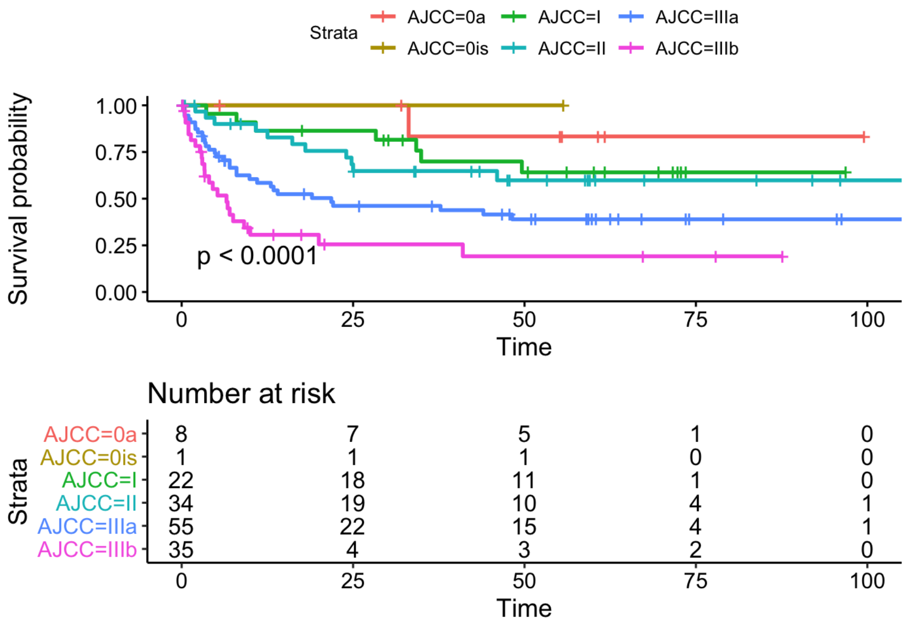

CSS

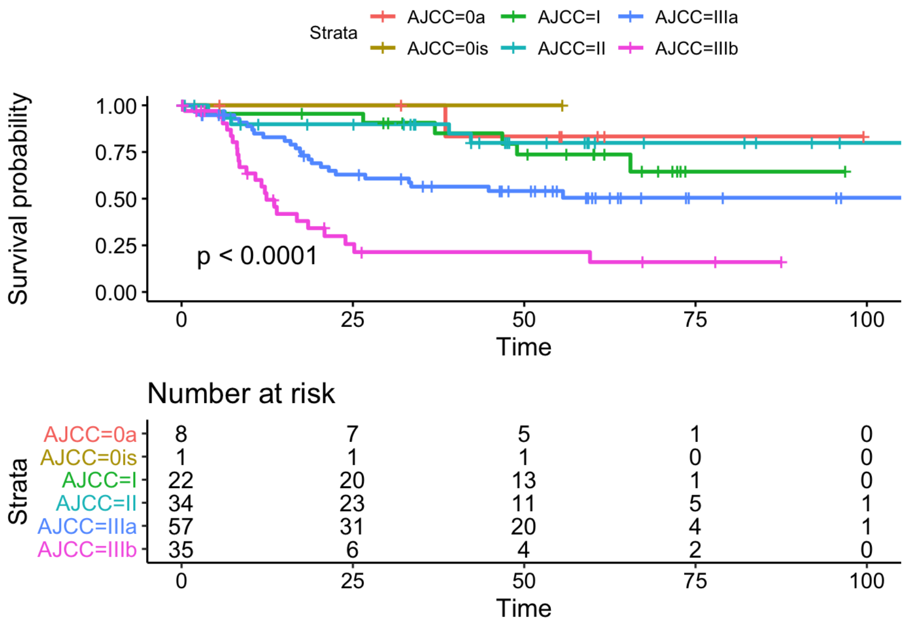

OS

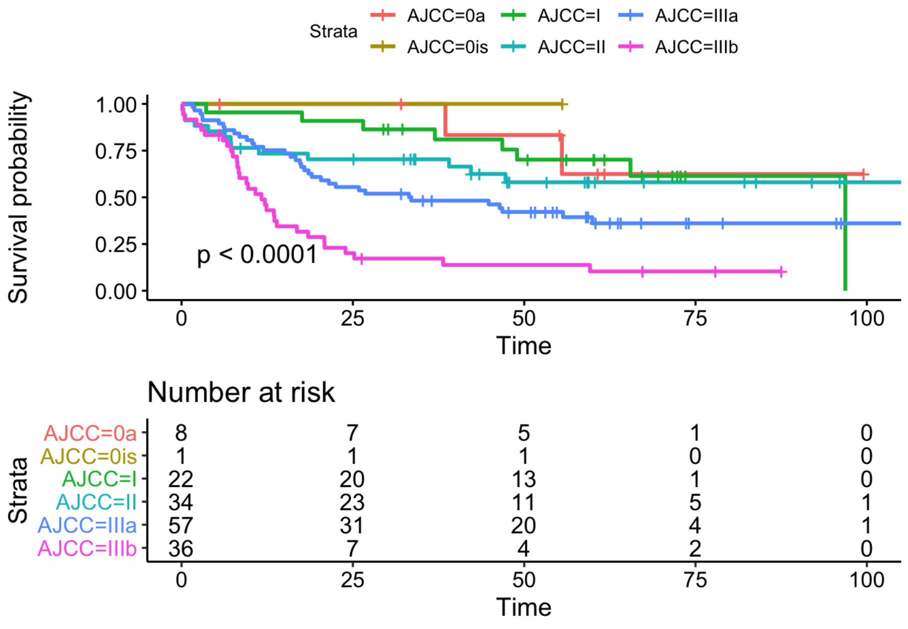

AJCC (simplified)

PFS

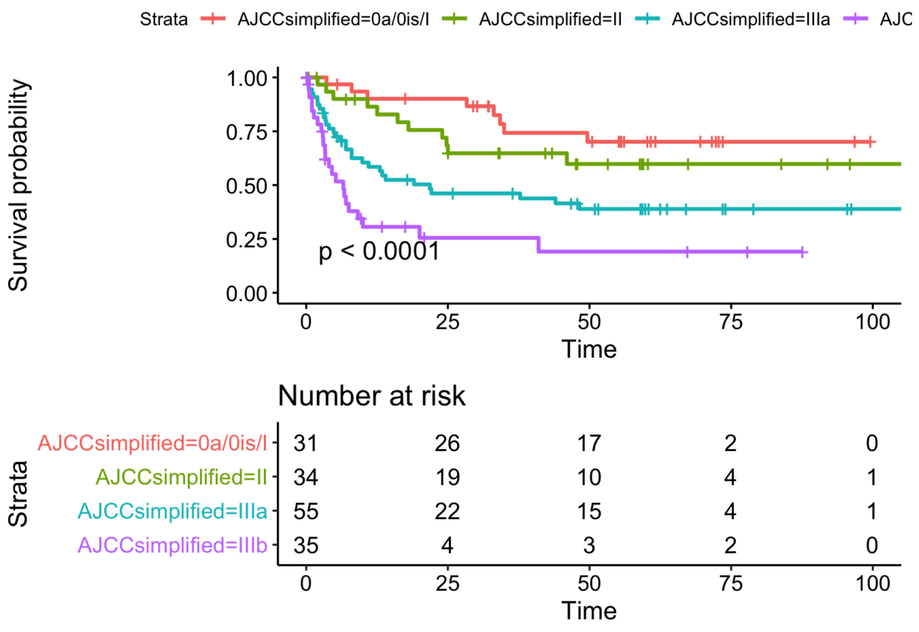

CSS

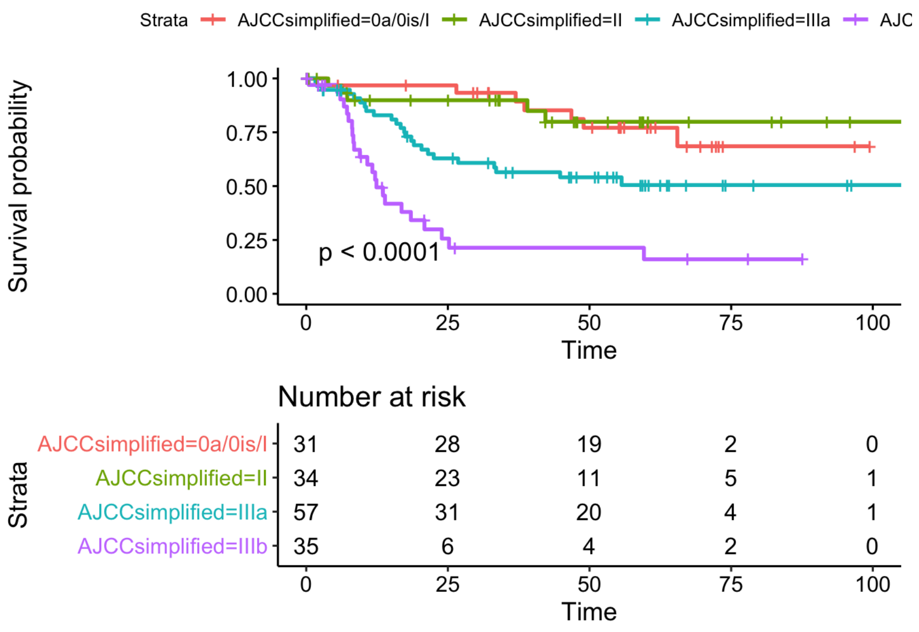

OS

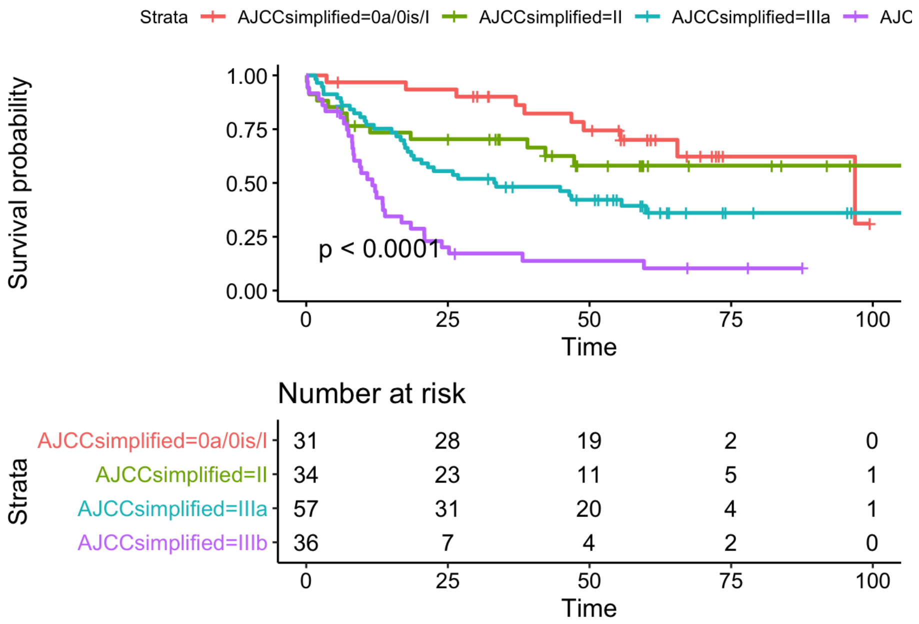

Exploration of immunoscore

Assessment of numeric behavior

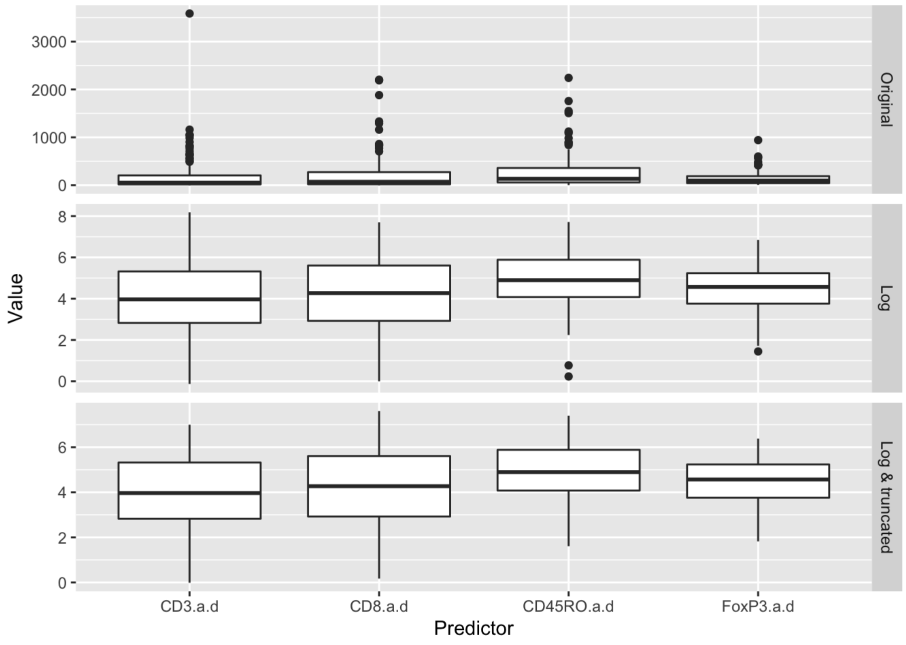

Log-transformed and truncated (1%, 99%) form of predictor shows normally-distributed behavior.

Assessment of collinearity

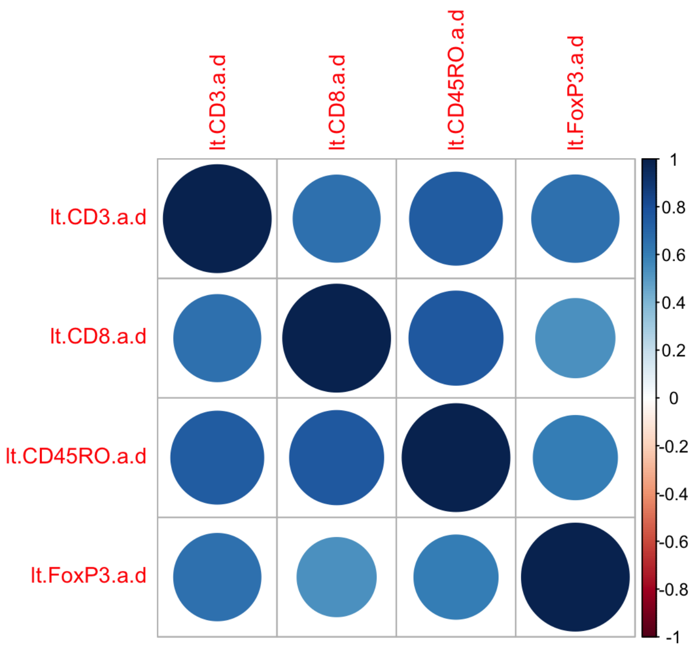

Unproblematic collinearity

Possible combinations

Single predictors

```
##             Combination Chisquared
## 1     PFS <= lt.CD3.a.d       3.33
## 2     PFS <= lt.CD8.a.d       7.87
## 3  PFS <= lt.CD45RO.a.d       7.51
## 4   PFS <= lt.FoxP3.a.d       8.55
## 5             PFS <= is       8.06
## 6     CSS <= lt.CD3.a.d       7.70
## 7     CSS <= lt.CD8.a.d      12.41
## 8  CSS <= lt.CD45RO.a.d       7.06
## 9   CSS <= lt.FoxP3.a.d      14.92
## 10            CSS <= is      12.91
## 11     OS <= lt.CD3.a.d       3.05
## 12     OS <= lt.CD8.a.d       5.39
## 13  OS <= lt.CD45RO.a.d       3.13
## 14   OS <= lt.FoxP3.a.d       8.27
## 15             OS <= is       5.82
```

lt.FoxP3.a.d. has the highest predictive potential. IS is a summary predictor representing the sum of log-transformed and truncated immunoscore components.

Combinations

```
##                                                     Combination Chisquared
## 1                                  PFS <= lt.CD3.a.d + lt.CD8.a.d       7.92
## 2                               PFS <= lt.CD3.a.d + lt.CD45RO.a.d       7.73
## 3                               PFS <= lt.CD8.a.d + lt.CD45RO.a.d       8.62
## 4                  PFS <= lt.CD3.a.d + lt.CD8.a.d + lt.CD45RO.a.d       9.17
## 5                                PFS <= lt.CD3.a.d + lt.FoxP3.a.d       8.58
## 6                                PFS <= lt.CD8.a.d + lt.FoxP3.a.d      10.42
## 7                   PFS <= lt.CD3.a.d + lt.CD8.a.d + lt.FoxP3.a.d      11.46
## 8                             PFS <= lt.CD45RO.a.d + lt.FoxP3.a.d       9.90
## 9                PFS <= lt.CD3.a.d + lt.CD45RO.a.d + lt.FoxP3.a.d      11.12
## 10               PFS <= lt.CD8.a.d + lt.CD45RO.a.d + lt.FoxP3.a.d      10.53
## 11 PFS <= lt.CD3.a.d + lt.CD8.a.d + lt.CD45RO.a.d + lt.FoxP3.a.d      12.17
## 12                                 CSS <= lt.CD3.a.d + lt.CD8.a.d      12.57
## 13                              CSS <= lt.CD3.a.d + lt.CD45RO.a.d       8.32
## 14                              CSS <= lt.CD8.a.d + lt.CD45RO.a.d      12.41
## 15                 CSS <= lt.CD3.a.d + lt.CD8.a.d + lt.CD45RO.a.d      12.66
## 16                               CSS <= lt.CD3.a.d + lt.FoxP3.a.d      15.02
## 17                               CSS <= lt.CD8.a.d + lt.FoxP3.a.d      17.49
## 18                  CSS <= lt.CD3.a.d + lt.CD8.a.d + lt.FoxP3.a.d      17.92
## 19                            CSS <= lt.CD45RO.a.d + lt.FoxP3.a.d      15.03
## 20               CSS <= lt.CD3.a.d + lt.CD45RO.a.d + lt.FoxP3.a.d      15.05
## 21               CSS <= lt.CD8.a.d + lt.CD45RO.a.d + lt.FoxP3.a.d      18.28
## 22 CSS <= lt.CD3.a.d + lt.CD8.a.d + lt.CD45RO.a.d + lt.FoxP3.a.d      18.36
## 23                                  OS <= lt.CD3.a.d + lt.CD8.a.d       7.76
## 24                               OS <= lt.CD3.a.d + lt.CD45RO.a.d       6.13
## 25                               OS <= lt.CD8.a.d + lt.CD45RO.a.d       8.04
## 26                  OS <= lt.CD3.a.d + lt.CD8.a.d + lt.CD45RO.a.d       8.09
## 27                                OS <= lt.CD3.a.d + lt.FoxP3.a.d      10.15
## 28                                OS <= lt.CD8.a.d + lt.FoxP3.a.d      11.51
## 29                   OS <= lt.CD3.a.d + lt.CD8.a.d + lt.FoxP3.a.d      12.19
## 30                             OS <= lt.CD45RO.a.d + lt.FoxP3.a.d      10.55
## 31                OS <= lt.CD3.a.d + lt.CD45RO.a.d + lt.FoxP3.a.d      10.92
## 32                OS <= lt.CD8.a.d + lt.CD45RO.a.d + lt.FoxP3.a.d      11.52
## 33  OS <= lt.CD3.a.d + lt.CD8.a.d + lt.CD45RO.a.d + lt.FoxP3.a.d      12.26
```

Combination of all components genearlly yields the highest chisquared value (4 degrees of freedom).

- PFS: 12.17 (2nd best: lt.CD3.a.d + lt.CD8.a.d + lt.FoxP3.a.d with 11.46)
- CSS: 18.36 (2nd best: lt.CD8.a.d + lt.CD45RO.a.d + lt.FoxP3.a.d with 18.28)
- OS: 12.26 (2nd best: lt.CD3.a.d + lt.CD8.a.d + lt.FoxP3.a.d with 12.19)

Reduction to three components (lt.CD3.a.d + lt.CD8.a.d + lt.FoxP3.a.d) would still yield reasonable chisquared values at 3 degrees of freedom (PFS: 12.17 instead of 11.46 (94%), CSS: 17.92 instead of 18.36 (98%), OS: 12.19 instead of 99%).

Quantification of the additional predictive benefit the immunoscore has on top of the already established AJCC classification

Univariable analysis

```
##                     PFS.HR PFS.LCL PFS.UCL PFS.P-value CSS.HR CSS.LCL CSS.UCL
## age                   1.00    0.98    1.03      0.7669   1.00    0.97    1.03
## gender                1.16    0.69    1.95      0.5805   1.17    0.65    2.11
## grade                 1.23    0.39    3.91      0.7266   0.99    0.31    3.18
## cis                   0.99    0.62    1.57      0.9546   1.21    0.73    2.02
## pne                   4.61    2.39    8.88      0.0000   4.58    2.27    9.21
## vi                    2.42    1.41    4.13      0.0013   2.71    1.55    4.74
## li                    3.50    2.19    5.58      0.0000   4.65    2.72    7.92
## AJCC0is               0.00    0.00     Inf      0.9954   0.00    0.00     Inf
## AJCCI                 2.61    0.32   21.18      0.3703   2.01    0.24   16.73
## AJCCII                3.32    0.43   25.70      0.2510   1.47    0.17   12.61
## AJCCIIIa              6.81    0.93   49.91      0.0591   4.41    0.60   32.65
## AJCCIIIb             13.98    1.88  104.07      0.0100  12.76    1.71   95.04
## AJCCsimplifiedII      1.59    0.64    3.95      0.3194   0.87    0.28    2.74
## AJCCsimplifiedIIIa    3.26    1.50    7.11      0.0029   2.60    1.12    6.05
## AJCCsimplifiedIIIb    6.69    2.96   15.14      0.0000   7.51    3.18   17.76
## lt.CD3.a.d            0.89    0.79    1.01      0.0690   0.82    0.72    0.95
## lt.CD8.a.d            0.82    0.72    0.94      0.0054   0.75    0.64    0.88
## lt.CD45RO.a.d         0.78    0.65    0.93      0.0063   0.77    0.63    0.93
## lt.FoxP3.a.d          0.74    0.60    0.91      0.0036   0.65    0.52    0.81
##                    CSS.P-value OS.HR OS.LCL OS.UCL OS.P-value
## age                     0.9467  1.01   0.99   1.04     0.3041
## gender                  0.5909  1.05   0.64   1.72     0.8385
## grade                   0.9929  1.16   0.42   3.17     0.7736
## cis                     0.4616  1.02   0.67   1.55     0.9381
## pne                     0.0000  3.51   1.97   6.24     0.0000
## vi                      0.0005  2.57   1.63   4.07     0.0001
## li                      0.0000  3.17   2.06   4.87     0.0000
## AJCC0is                 0.9964  0.00   0.00    Inf     0.9951
## AJCCI                   0.5172  1.41   0.30   6.67     0.6622
## AJCCII                  0.7236  2.01   0.45   8.93     0.3591
## AJCCIIIa                0.1458  3.28   0.78  13.70     0.1038
## AJCCIIIb                0.0130  8.07   1.91  34.09     0.0045
## AJCCsimplifiedII        0.8092  1.59   0.70   3.64     0.2685
## AJCCsimplifiedIIIa      0.0263  2.60   1.28   5.27     0.0082
## AJCCsimplifiedIIIb      0.0000  6.39   3.09  13.23     0.0000
## lt.CD3.a.d              0.0060  0.90   0.81   1.01     0.0813
## lt.CD8.a.d              0.0005  0.86   0.76   0.98     0.0208
## lt.CD45RO.a.d           0.0082  0.86   0.73   1.02     0.0772
## lt.FoxP3.a.d            0.0001  0.76   0.63   0.92     0.0042
```

Interpretation:

- As expected, we see that the AJCC stages are a good tool for risk stratificiation.
- We further see the negative effects of pne, vi and li.
- Immunoscore components have a protective effect (mostly all of them are statistically significant/borderline significant).

Next step: multivariable models

Multivariable analysis

AJCCsimplified model

```
## [[1]]
##                     PFS.HR PFS.LCL PFS.UCL PFS.P-value CSS.HR CSS.LCL CSS.UCL
## AJCCsimplifiedII      1.59    0.64    3.95      0.3194   0.87    0.28    2.74
## AJCCsimplifiedIIIa    3.26    1.50    7.11      0.0029   2.60    1.12    6.05
## AJCCsimplifiedIIIb    6.69    2.96   15.14      0.0000   7.51    3.18   17.76
##                    CSS.P-value OS.HR OS.LCL OS.UCL OS.P-value
## AJCCsimplifiedII        0.8092  1.59   0.70   3.64     0.2685
## AJCCsimplifiedIIIa      0.0263  2.60   1.28   5.27     0.0082
## AJCCsimplifiedIIIb      0.0000  6.39   3.09  13.23     0.0000
##
## [[2]]
## Concordance PFS Concordance CSS  Concordance OS
##       0.6931002       0.7112978       0.6742863
```

AJCCsimplified + CD3 model

```
## [[1]]
##                    PFS.HR PFS.LCL PFS.UCL PFS.P-value CSS.HR CSS.LCL CSS.UCL
## AJCCsimplifiedII     1.68    0.67    4.17      0.2676   0.92    0.29    2.91
## AJCCsimplifiedIIIa   3.44    1.58    7.51      0.0019   2.70    1.16    6.29
## AJCCsimplifiedIIIb   6.88    3.04   15.61      0.0000   7.57    3.19   17.94
## lt.CD3.a.d           0.89    0.78    1.00      0.0497   0.83    0.72    0.96
##                    CSS.P-value OS.HR OS.LCL OS.UCL OS.P-value
## AJCCsimplifiedII        0.8924  1.64   0.72   3.74     0.2407
## AJCCsimplifiedIIIa      0.0209  2.67   1.31   5.42     0.0067
## AJCCsimplifiedIIIb      0.0000  6.45   3.11  13.36     0.0000
## lt.CD3.a.d              0.0101  0.90   0.81   1.01     0.0875
##
## [[2]]
## Concordance PFS Concordance CSS  Concordance OS
##       0.7152353       0.7450593       0.6894183
```

AJCCsimplified + CD8 model

```
## [[1]]
##                    PFS.HR PFS.LCL PFS.UCL PFS.P-value CSS.HR CSS.LCL CSS.UCL
## AJCCsimplifiedII     1.68    0.67    4.17      0.2675   0.91    0.29    2.86
## AJCCsimplifiedIIIa   3.32    1.52    7.25      0.0025   2.58    1.11    6.01
## AJCCsimplifiedIIIb   6.05    2.66   13.79      0.0000   6.49    2.72   15.50
## lt.CD8.a.d           0.87    0.76    1.00      0.0457   0.82    0.70    0.96
##                    CSS.P-value OS.HR OS.LCL OS.UCL OS.P-value
## AJCCsimplifiedII        0.8671  1.63   0.71   3.71     0.2479
## AJCCsimplifiedIIIa      0.0276  2.61   1.28   5.30     0.0080
## AJCCsimplifiedIIIb      0.0000  6.03   2.89  12.54     0.0000
## lt.CD8.a.d              0.0157  0.91   0.80   1.04     0.1562
##
## [[2]]
## Concordance PFS Concordance CSS  Concordance OS
##       0.7079234       0.7394598       0.6812621
```

AJCCsimplified + CD45RO model

```
## [[1]]
##                    PFS.HR PFS.LCL PFS.UCL PFS.P-value CSS.HR CSS.LCL CSS.UCL
## AJCCsimplifiedII     1.76    0.70    4.40      0.2258   0.94    0.30    2.96
## AJCCsimplifiedIIIa   3.35    1.54    7.30      0.0024   2.59    1.11    6.02
## AJCCsimplifiedIIIb   6.46    2.85   14.64      0.0000   7.06    2.98   16.73
## lt.CD45RO.a.d        0.80    0.66    0.97      0.0217   0.82    0.67    1.02
##                    CSS.P-value OS.HR OS.LCL OS.UCL OS.P-value
## AJCCsimplifiedII        0.9111  1.65   0.72   3.78     0.2353
## AJCCsimplifiedIIIa      0.0273  2.60   1.28   5.28     0.0081
## AJCCsimplifiedIIIb      0.0000  6.22   3.00  12.90     0.0000
## lt.CD45RO.a.d           0.0745  0.91   0.76   1.08     0.2774
##
## [[2]]
## Concordance PFS Concordance CSS  Concordance OS
##       0.7093858       0.7318841       0.6780425
```

AJCCsimplified + FoxP3 model

```
## [[1]]
##                    PFS.HR PFS.LCL PFS.UCL PFS.P-value CSS.HR CSS.LCL CSS.UCL
## AJCCsimplifiedII     2.15    0.85    5.48      0.1069   1.37    0.42    4.47
## AJCCsimplifiedIIIa   3.96    1.80    8.74      0.0006   3.22    1.37    7.56
## AJCCsimplifiedIIIb   7.62    3.35   17.36      0.0000   9.16    3.82   21.95
## lt.FoxP3.a.d         0.70    0.57    0.87      0.0012   0.62    0.49    0.80
##                    CSS.P-value OS.HR OS.LCL OS.UCL OS.P-value
## AJCCsimplifiedII        0.5981  2.11   0.90   4.92     0.0844
## AJCCsimplifiedIIIa      0.0074  2.98   1.46   6.09     0.0028
## AJCCsimplifiedIIIb      0.0000  7.20   3.45  15.00     0.0000
## lt.FoxP3.a.d            0.0001  0.72   0.59   0.89     0.0019
##
## [[2]]
## Concordance PFS Concordance CSS  Concordance OS
##       0.7353762       0.7677866       0.7088431
```

AJCCsimplified + CD3 + CD8 + FoxP3 model (three components)

```
## [[1]]
##                    PFS.HR PFS.LCL PFS.UCL PFS.P-value CSS.HR CSS.LCL CSS.UCL
## AJCCsimplifiedII     2.16    0.84    5.53      0.1083   1.33    0.40    4.36
## AJCCsimplifiedIIIa   3.95    1.78    8.75      0.0007   3.15    1.33    7.44
## AJCCsimplifiedIIIb   7.51    3.23   17.46      0.0000   8.89    3.64   21.71
## lt.CD3.a.d           1.02    0.84    1.23      0.8581   0.98    0.80    1.21
## lt.CD8.a.d           0.98    0.81    1.20      0.8669   0.97    0.78    1.22
## lt.FoxP3.a.d         0.70    0.52    0.95      0.0199   0.65    0.47    0.91
##                    CSS.P-value OS.HR OS.LCL OS.UCL OS.P-value
## AJCCsimplifiedII        0.6403  2.15   0.91   5.07     0.0795
## AJCCsimplifiedIIIa      0.0090  3.01   1.47   6.18     0.0026
## AJCCsimplifiedIIIb      0.0000  7.33   3.48  15.44     0.0000
## lt.CD3.a.d              0.8509  1.01   0.85   1.20     0.8950
## lt.CD8.a.d              0.8140  1.02   0.85   1.22     0.8324
## lt.FoxP3.a.d            0.0110  0.70   0.53   0.93     0.0121
##
## [[2]]
## Concordance PFS Concordance CSS  Concordance OS
##       0.7363733       0.7689394       0.7103456
```

AJCCsimplified + CD3 + CD8 + CD45RO model (three components)

```
## [[1]]
##                    PFS.HR PFS.LCL PFS.UCL PFS.P-value CSS.HR CSS.LCL CSS.UCL
## AJCCsimplifiedII     1.75    0.70    4.37      0.2311   0.90    0.29    2.86
## AJCCsimplifiedIIIa   3.36    1.54    7.35      0.0023   2.69    1.15    6.28
## AJCCsimplifiedIIIb   6.35    2.76   14.59      0.0000   7.21    2.98   17.47
## lt.CD3.a.d           0.99    0.80    1.22      0.9074   0.84    0.66    1.06
## lt.CD8.a.d           0.96    0.77    1.19      0.6921   0.88    0.70    1.10
## lt.CD45RO.a.d        0.85    0.60    1.21      0.3637   1.15    0.79    1.67
##                    CSS.P-value OS.HR OS.LCL OS.UCL OS.P-value
## AJCCsimplifiedII        0.8636  1.61   0.71   3.69     0.2572
## AJCCsimplifiedIIIa      0.0218  2.68   1.32   5.46     0.0065
## AJCCsimplifiedIIIb      0.0000  6.44   3.07  13.54     0.0000
## lt.CD3.a.d              0.1394  0.89   0.73   1.08     0.2347
## lt.CD8.a.d              0.2489  0.95   0.78   1.14     0.5761
## lt.CD45RO.a.d           0.4692  1.10   0.81   1.51     0.5338
##
## [[2]]
## Concordance PFS Concordance CSS  Concordance OS
##       0.7101835       0.7485178       0.6908135
```

AJCCsimplified + CD3 + CD8 + lt.CD45R0 + FoxP3 model (all components)

```
## [[1]]
##                     PFS.HR PFS.LCL PFS.UCL PFS.P-value CSS.HR CSS.LCL CSS.UCL
## AJCCsimplifiedII      2.24    0.87    5.76      0.0947   1.29    0.39    4.23
## AJCCsimplifiedIIIa    3.93    1.77    8.71      0.0008   3.21    1.36    7.58
## AJCCsimplifiedIIIb    7.53    3.24   17.50      0.0000   9.30    3.77   22.92
## lt.CD3.a.d            1.07    0.86    1.35      0.5422   0.91    0.71    1.16
## lt.CD8.a.d            1.03    0.82    1.28      0.8262   0.94    0.74    1.18
## lt.CD45RO.a.d         0.86    0.60    1.23      0.3957   1.23    0.83    1.83
## lt.FoxP3.a.d          0.70    0.52    0.95      0.0208   0.64    0.46    0.89
##                     CSS.P-value OS.HR OS.LCL OS.UCL OS.P-value
## AJCCsimplifiedII         0.6754  2.11   0.89   4.97     0.0882
## AJCCsimplifiedIIIa       0.0079  3.05   1.49   6.27     0.0023
## AJCCsimplifiedIIIb       0.0000  7.56   3.57  16.02     0.0000
## lt.CD3.a.d               0.4373  0.96   0.78   1.18     0.6828
## lt.CD8.a.d               0.5732  0.99   0.82   1.20     0.9014
## lt.CD45RO.a.d            0.2926  1.16   0.84   1.60     0.3681
## lt.FoxP3.a.d             0.0080  0.69   0.53   0.91     0.0096
##
## [[2]]
## Concordance PFS Concordance CSS  Concordance OS
##       0.7305238       0.7717391       0.7133505
```

Optimism-corrected concordance: AJCCsimplified + CD3 + CD8 + lt.CD45R0 + FoxP3 model (all components)

Interpretation:

- Among the multivariable models incorporating AJCCsimplified and one immunoscore component: immunoscore component remains significant
- Among the multivariable models incorporating AJCCsimplified and three or all immunoscore components: not significant anymore (probably due to sample size)
- Addition of immunoscore components considerably increases the concordance index of the AJCCsimplified-only model, even after correcting for overfitting.

Outcome	AJCCsimplified	Plus Immunoscore (bootstrapped)
PFS	0.69	0.73 (0.71)
CSS	0.71	0.77 (0.76)
OS	0.67	0.71 (0.70)

Next step: Is it helpful to substratify AJCC stages according to immunoscore? [e.g., “high immunoscore / favourable risk”, “low immunoscore / unfavourable risk”]

The utility of the immunoscore to substratify patients within AJCC groups

Full cohort

All patients were used to develop the immunoscore model. The model was then used to stratify all patients into “high immunoscore / favourable risk” and “low immunoscore / unfavourable risk”.

Kaplan-Meier curves

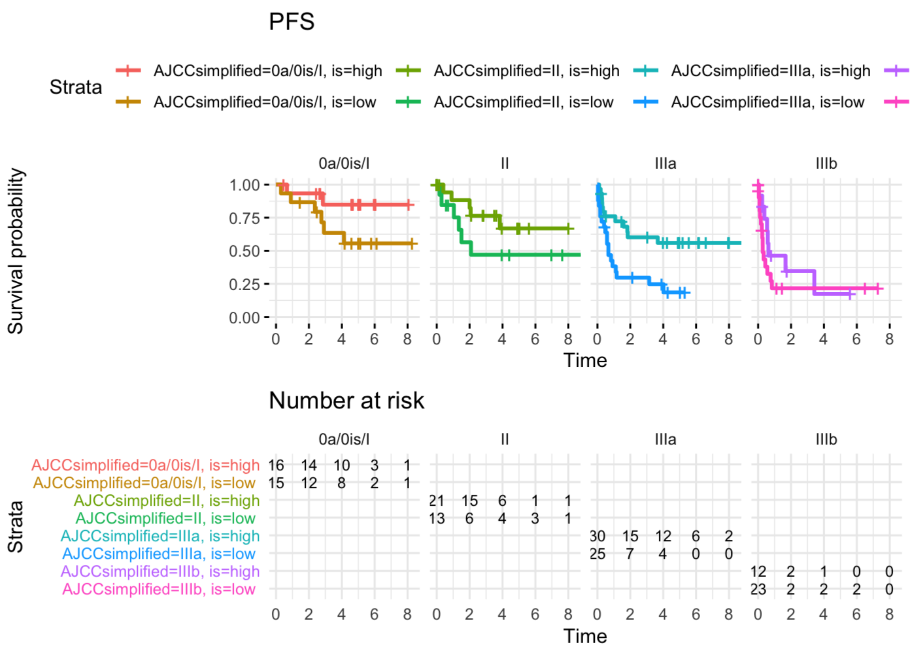

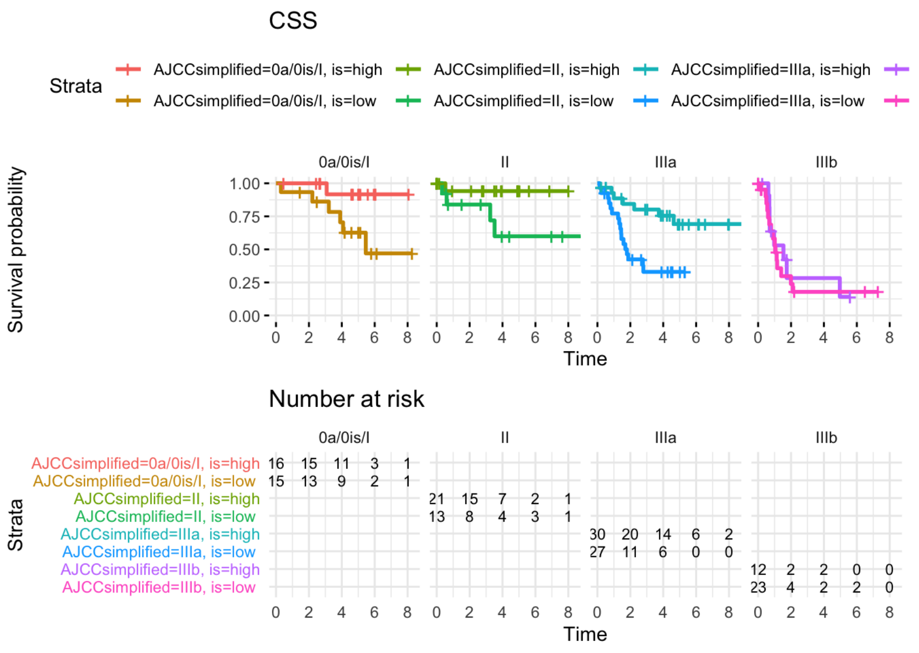

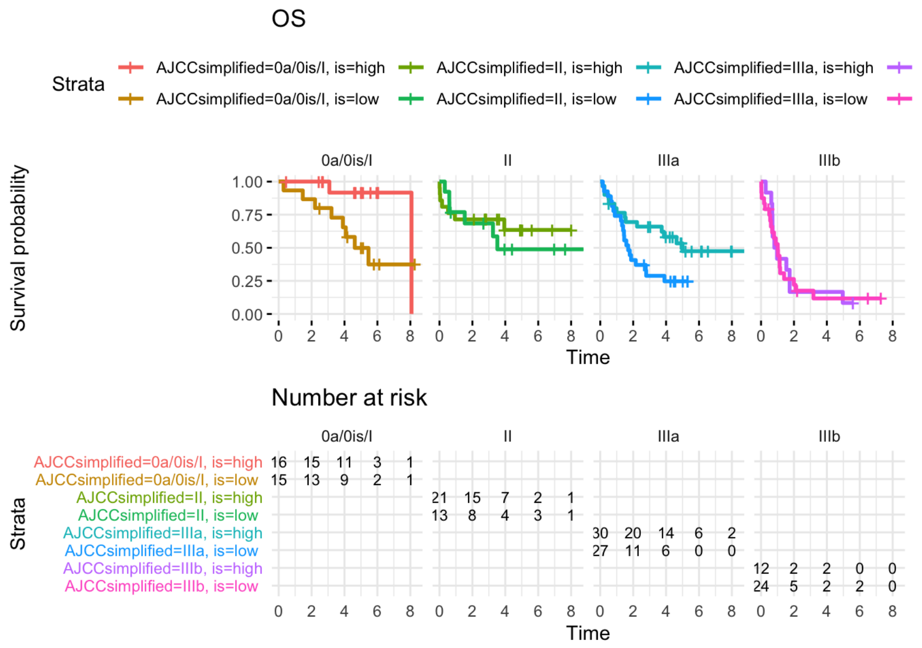

Kaplan-Meier curves (modified)

```
## quartz_off_screen
##                  2
```

```
## quartz_off_screen
##                  2
```

```
## quartz_off_screen
##                  2
```

```
## quartz_off_screen
##                  2
```

```
## quartz_off_screen
##                  2
```

```
## quartz_off_screen
##                  2
```

```
## quartz_off_screen
##                  2
```

```
## quartz_off_screen
##                  2
```

```
## quartz_off_screen
##                  2
```

```
## quartz_off_screen
##                  2
```

```
## quartz_off_screen
##                  2
```

```
## quartz_off_screen
##                  2
```

```
## quartz_off_screen
##                  2
```

```
## quartz_off_screen
##                  2
```

```
## quartz_off_screen
##                  2
```

Associated hazard ratios (Cox proportional hazards regression)

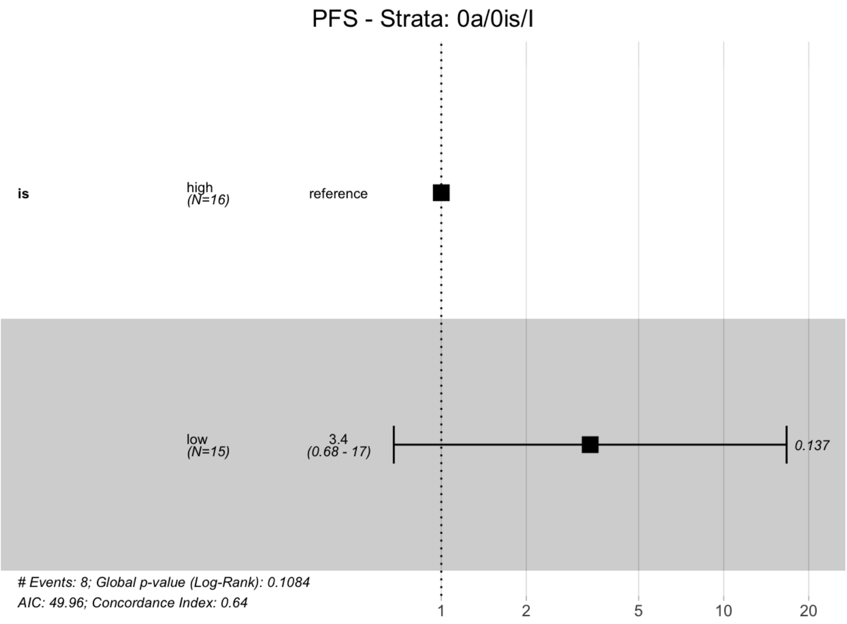
PFS - Strata: 0a/0is/I
is
high
(N=16)
reference
low
(N=15)
3.4
(0.68 - 17)
0.137
Events: 8; Global p-value (Log-Rank): 0.1084
AIC: 49.96; Concordance Index: 0.64
1
2
5
10
20

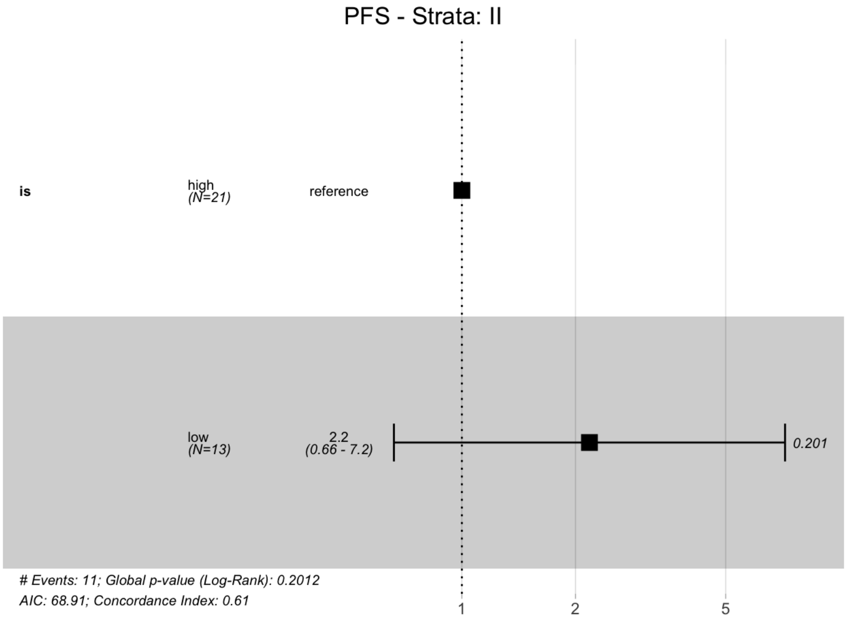
PFS - Strata: II
is
high
(N=21)
reference
low
(N=13)
2.2
(0.66 - 7.2)
0.201
Events: 11; Global p-value (Log-Rank): 0.2012
AIC: 68.91; Concordance Index: 0.61
1
2
5

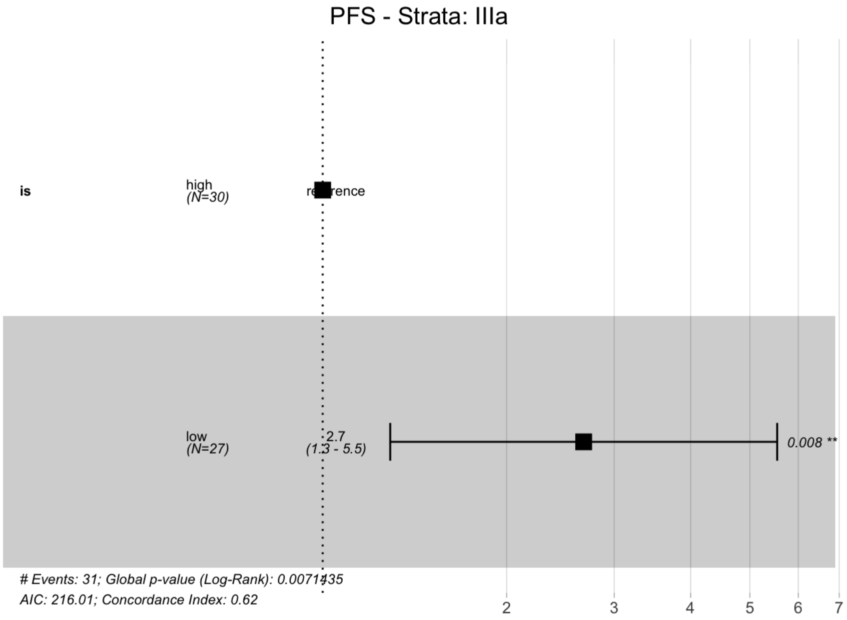
PFS - Strata: IIIa
is
high
(N=30)
reference
low
(N=27)
2.7
(1.3 - 5.5)
0.008 **
Events: 31; Global p-value (Log-Rank): 0.0071435
AIC: 216.01; Concordance Index: 0.62
2
3
4
5
6
7

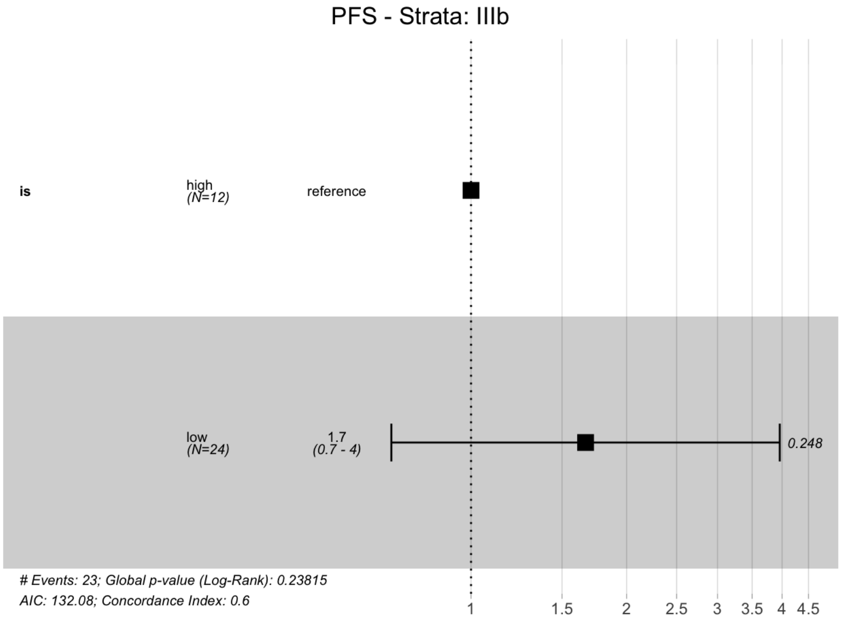
PFS - Strata: IIIb
is
high
(N=12)
reference
low
(N=24)
1.7
(0.7 - 4)
0.248
Events: 23; Global p-value (Log-Rank): 0.23815
AIC: 132.08; Concordance Index: 0.6
1
1.5
2
2.5
3
3.5
4
4.5

CSS - Strata: 0a/0is/I

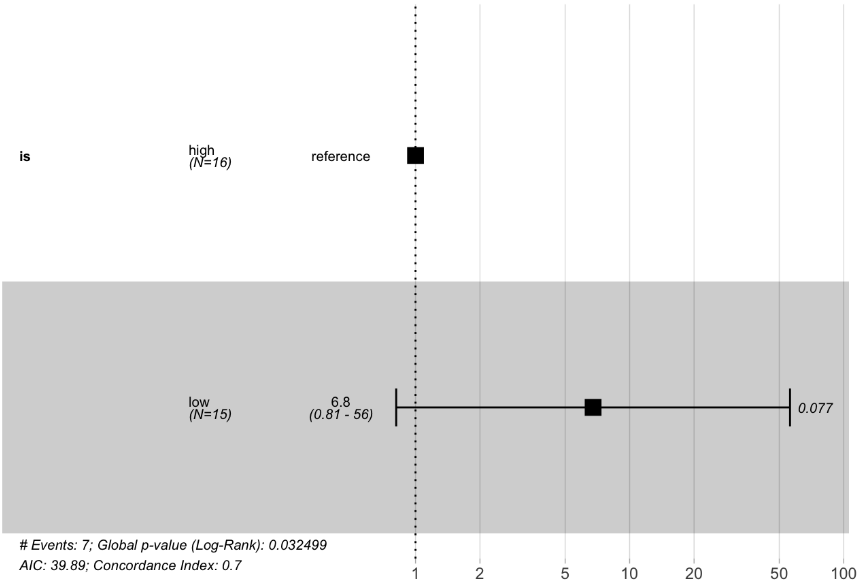

CSS - Strata: II

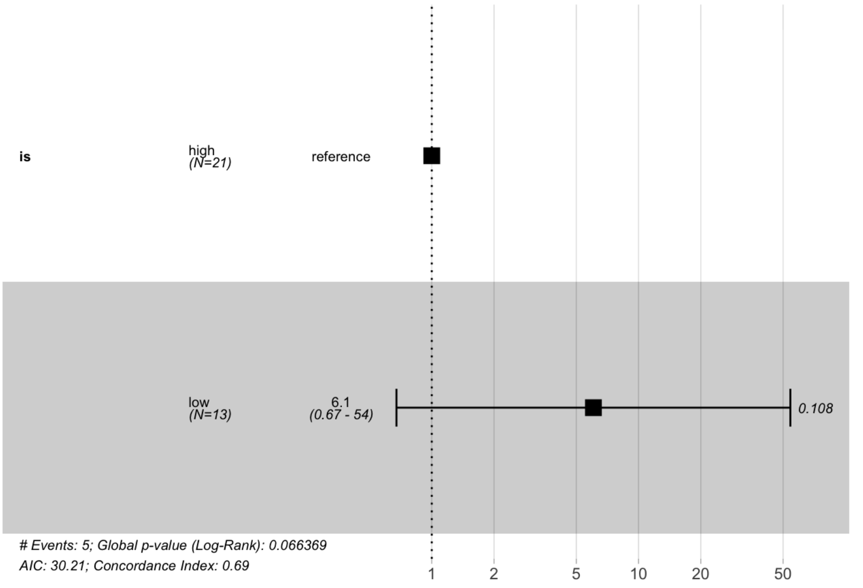

CSS - Strata: IIIa

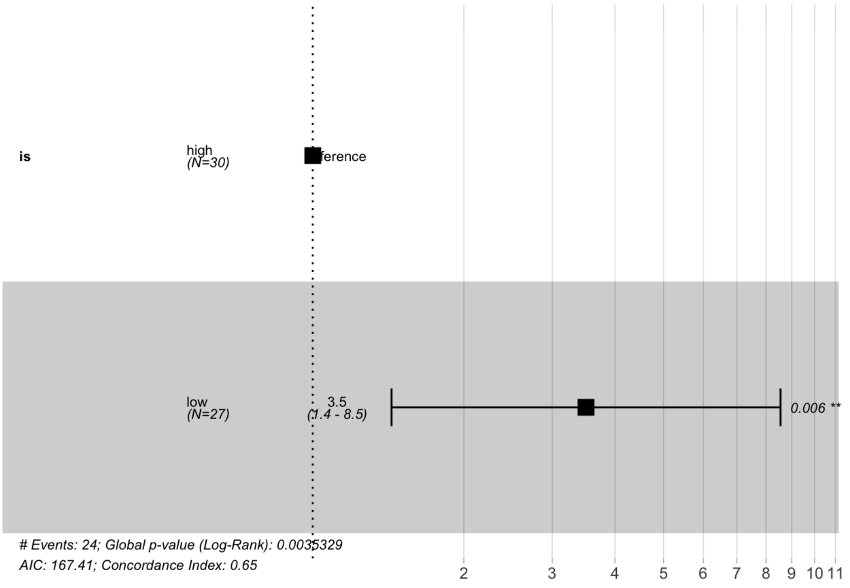

CSS - Strata: IIIb

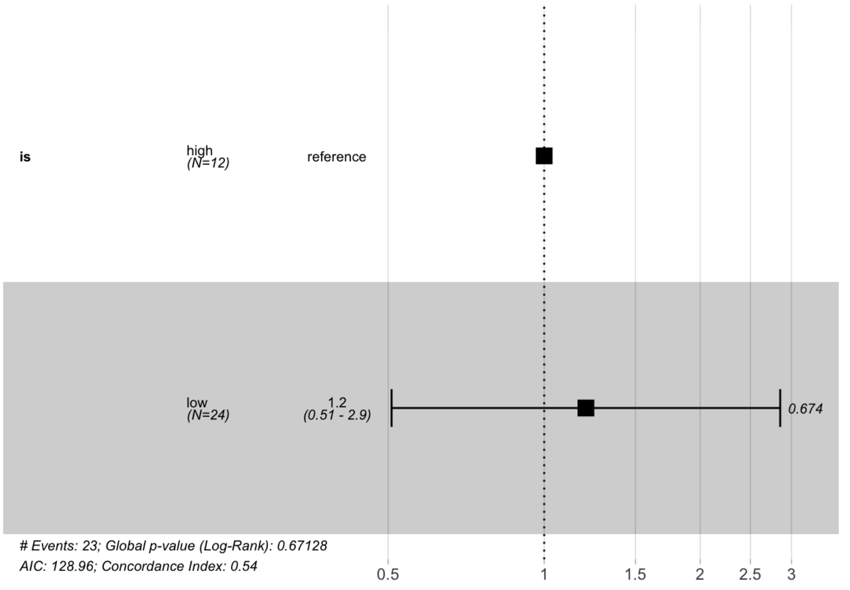

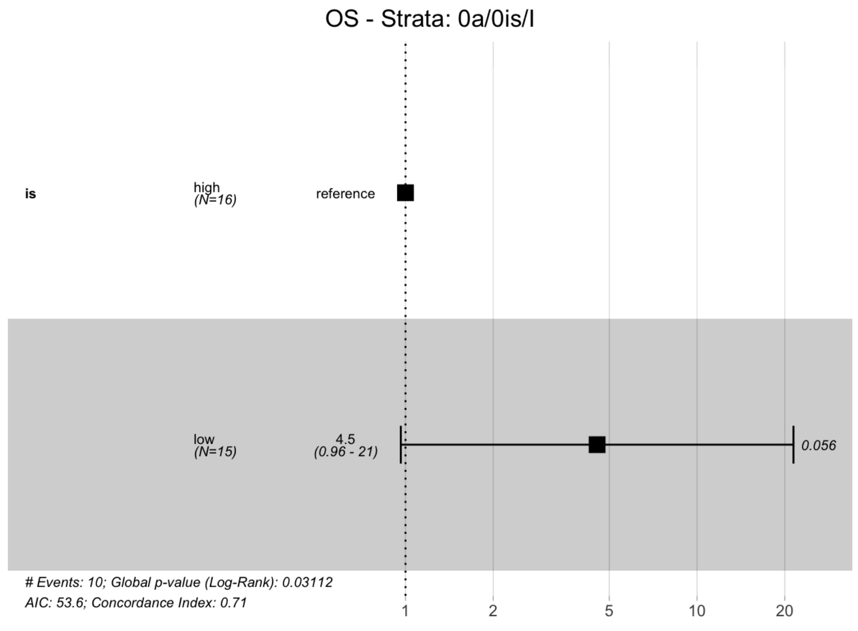

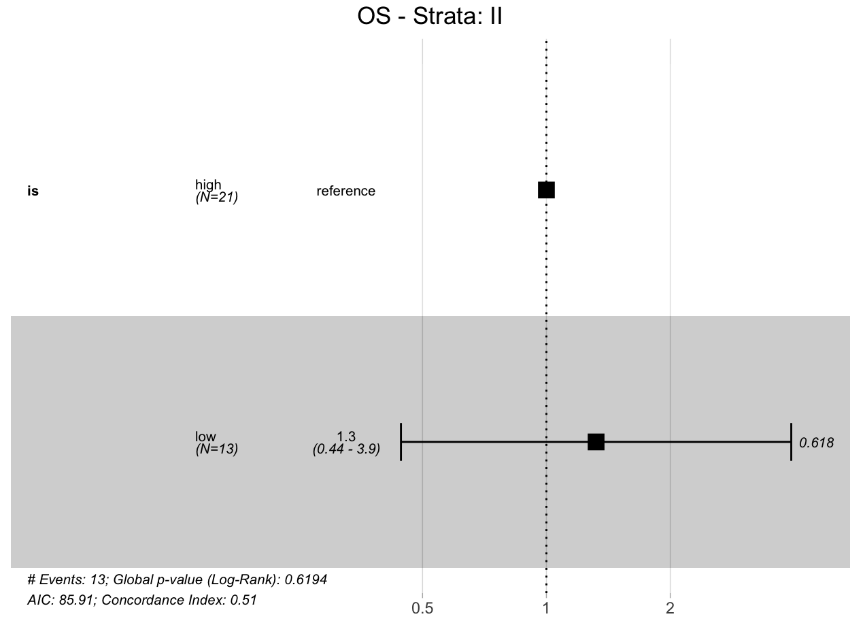

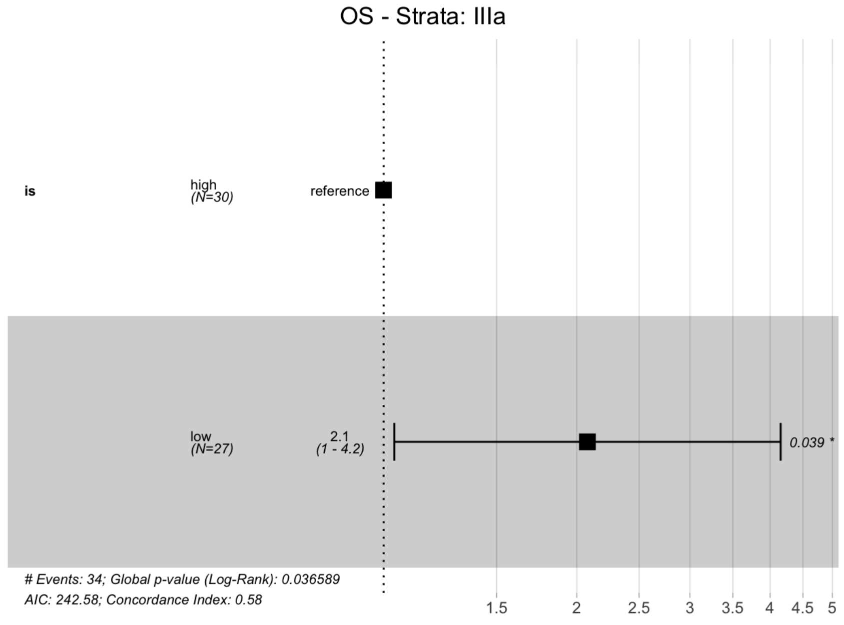

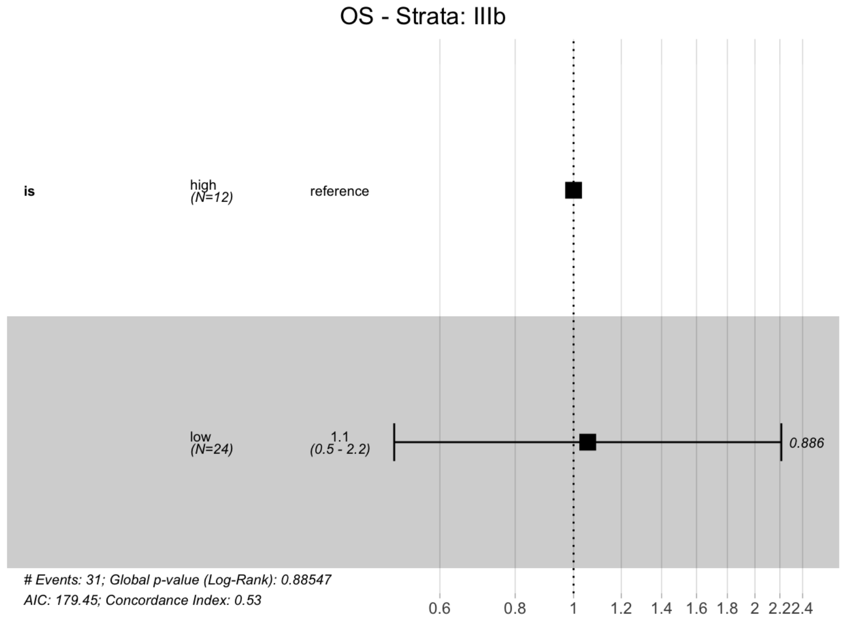

Split sample approach (external validation)

2/3 of all patients were randomly selected to be the development cohort in which the immunoscore model was developed while the remaining patients served as validation cohort in which we used the model to stratify all patients into “high immunoscore / favourable risk” and “low immunoscore / unfavourable risk”.

Kaplan-Meier curves

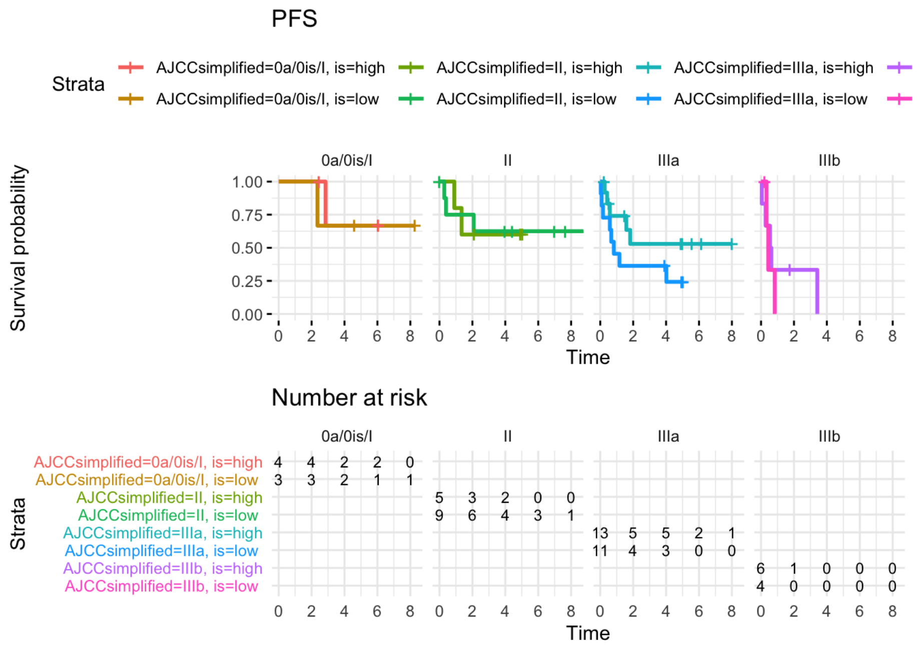

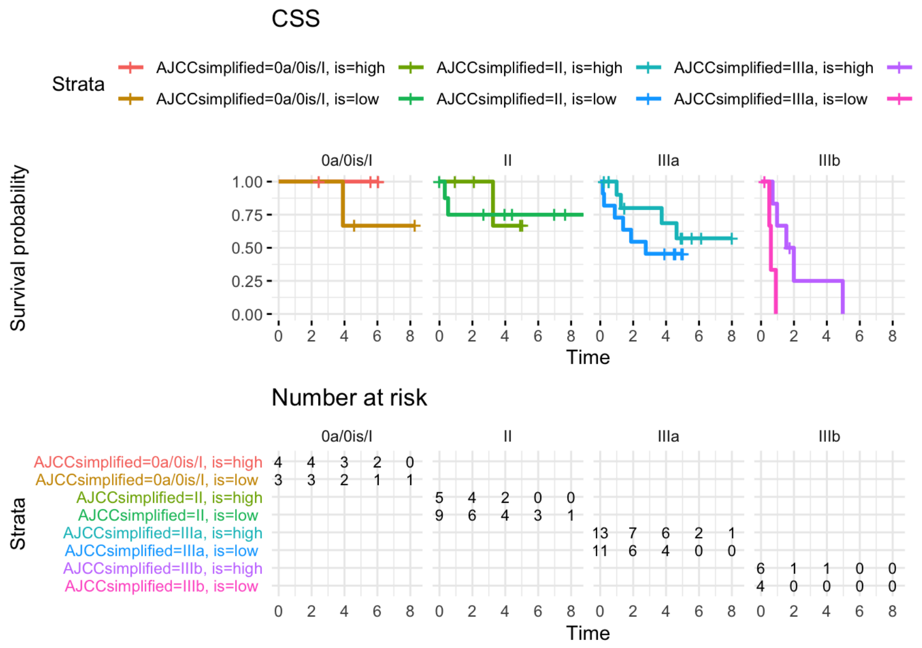

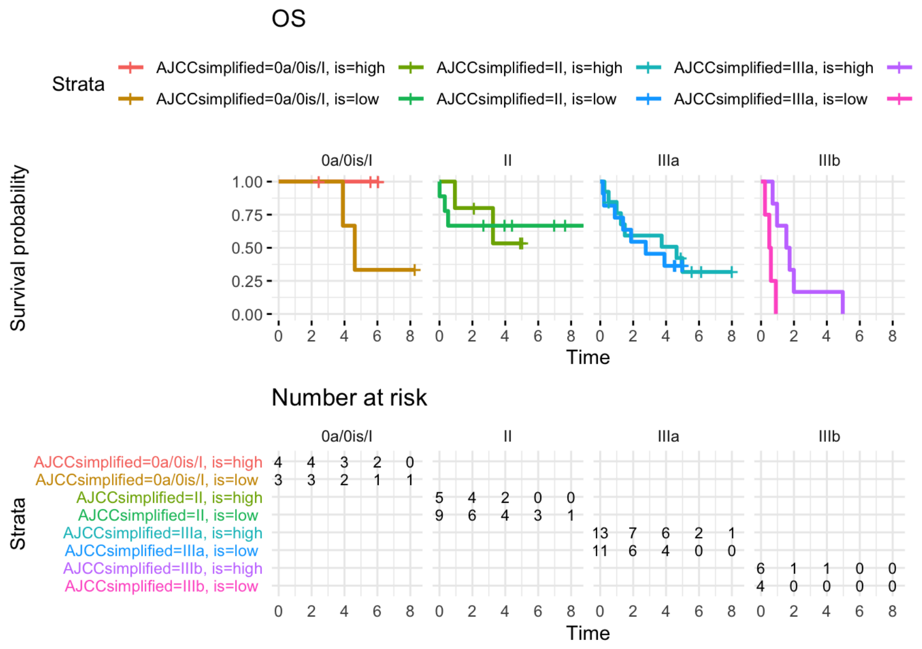

Associated hazard ratios (Cox proportional hazards regression)

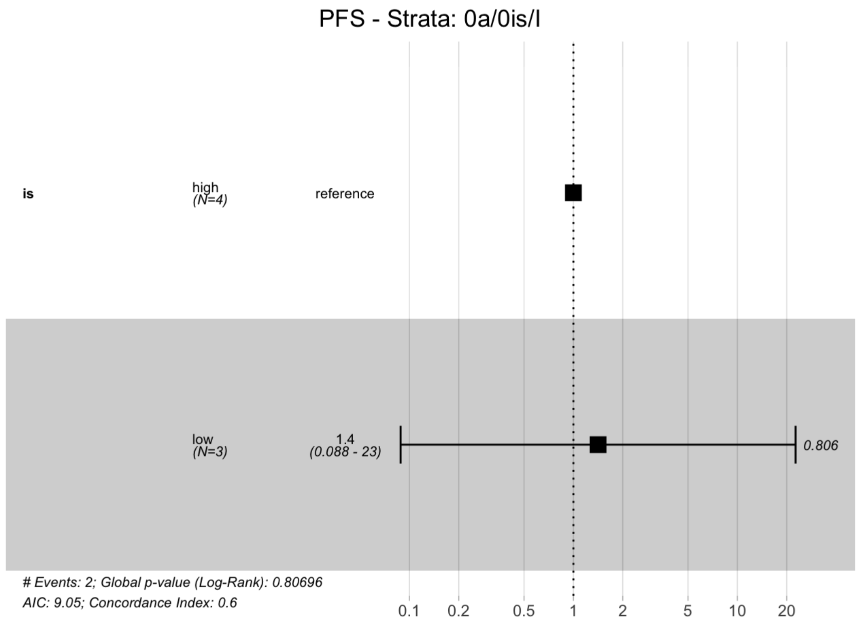
PFS - Strata: 0a/0is/I
is
high
(N=4)
reference
low
(N=3)
1.4
(0.088 - 23)
0.806
Events: 2; Global p-value (Log-Rank): 0.80696
AIC: 9.05; Concordance Index: 0.6
0.1 0.2 0.5 1 2 5 10 20

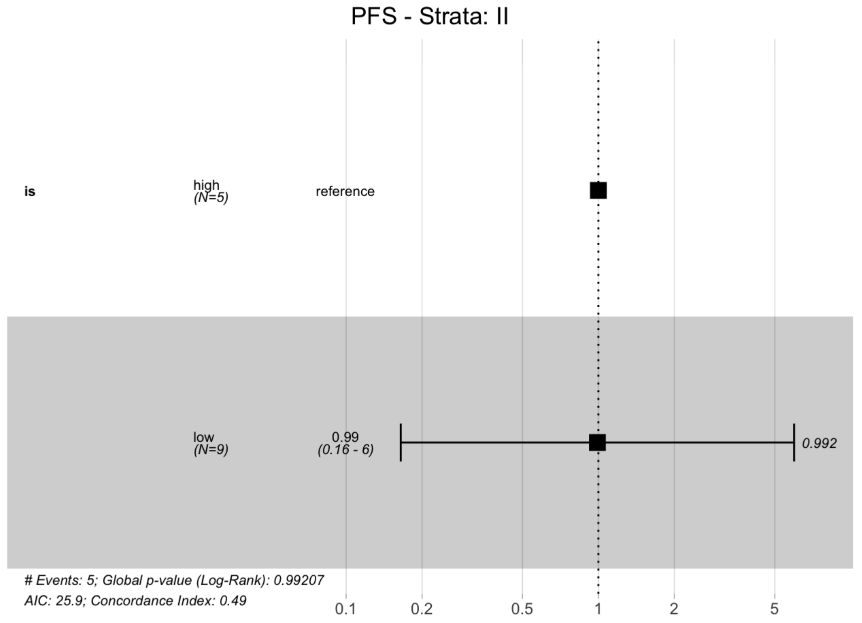
PFS - Strata: II
is
high
(N=5)
reference
low
(N=9)
0.99
(0.16 - 6)
0.992
Events: 5; Global p-value (Log-Rank): 0.99207
AIC: 25.9; Concordance Index: 0.49
0.1 0.2 0.5 1 2 5

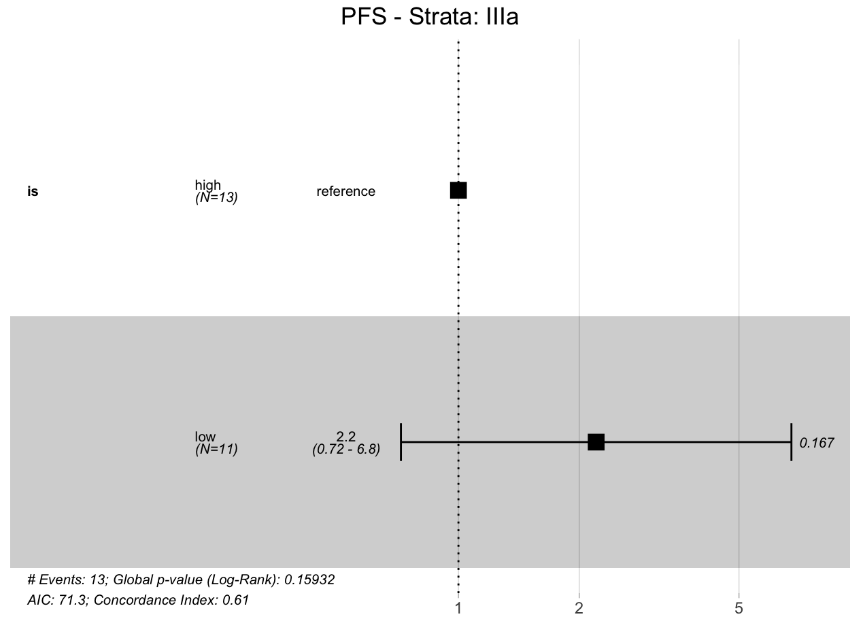
PFS - Strata: IIIa
is
high
(N=13)
reference
low
(N=11)
2.2
(0.72 - 6.8)
0.167
Events: 13; Global p-value (Log-Rank): 0.15932
AIC: 71.3; Concordance Index: 0.61
1 2 5

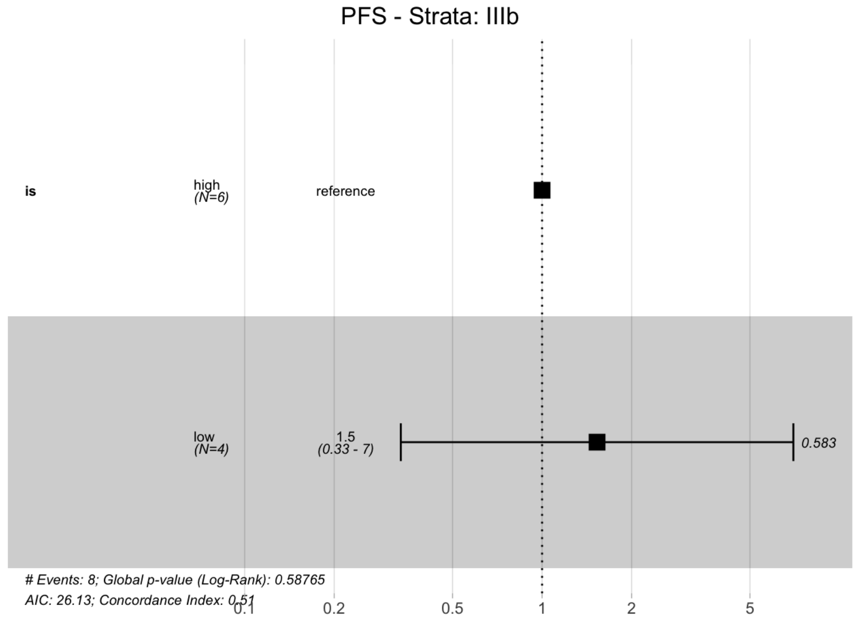
PFS - Strata: IIIb
is
high
(N=6)
reference
low
(N=4)
1.5
(0.33 - 7)
0.583
Events: 8; Global p-value (Log-Rank): 0.58765
AIC: 26.13; Concordance Index: 0.51
0.1 0.2 0.5 1 2 5

CSS - Strata: 0a/0is/I

CSS - Strata: II

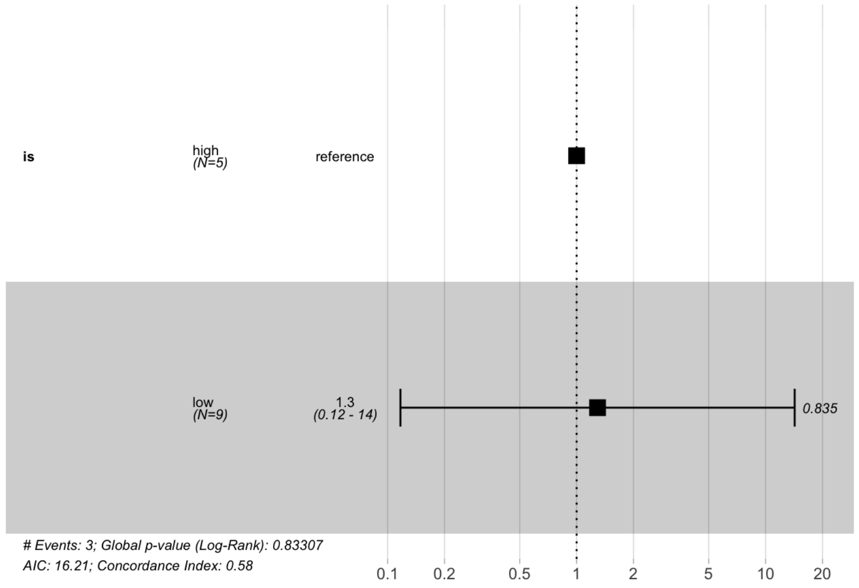

CSS - Strata: IIIa

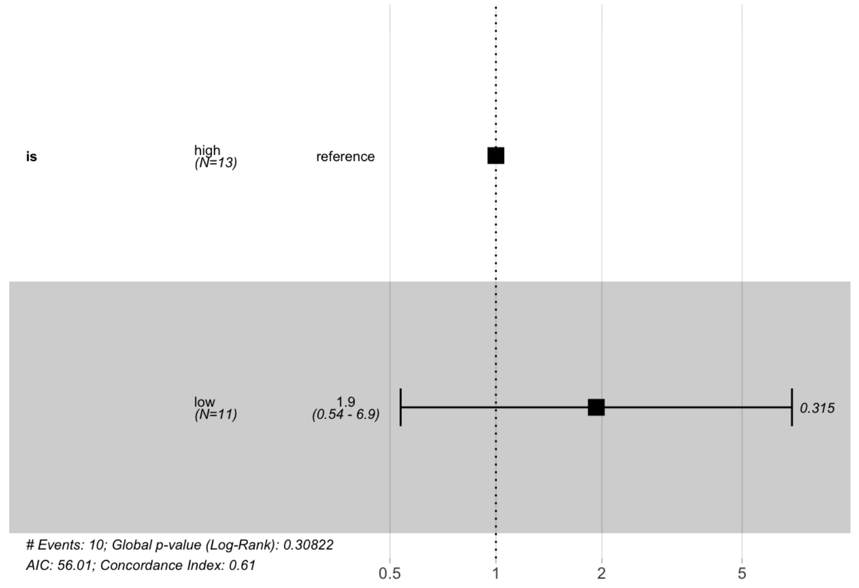

CSS - Strata: IIIb

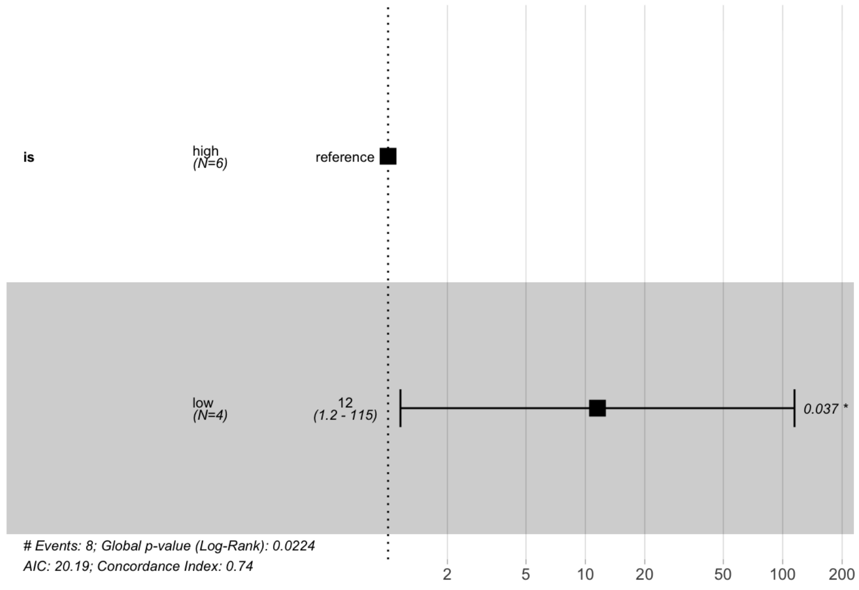

OS - Strata: 0a/0is/I

OS - Strata: II

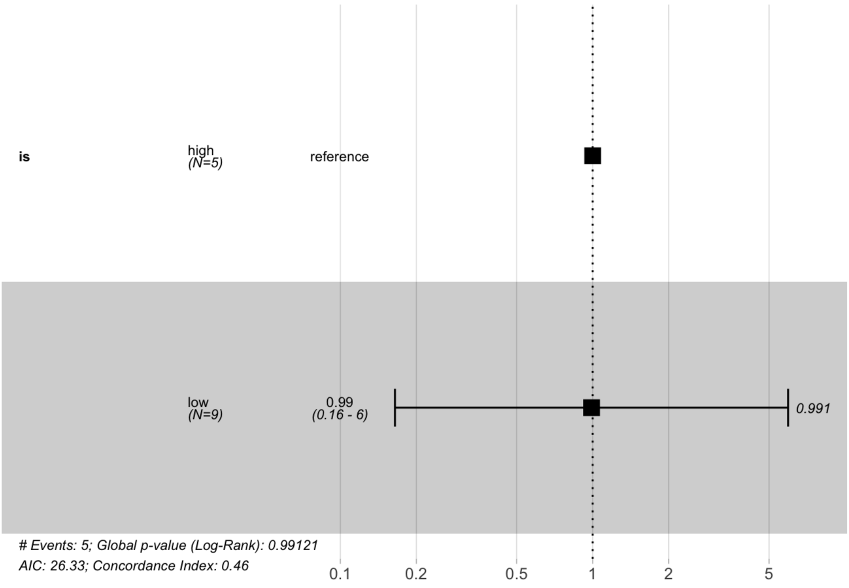

OS - Strata: IIIa

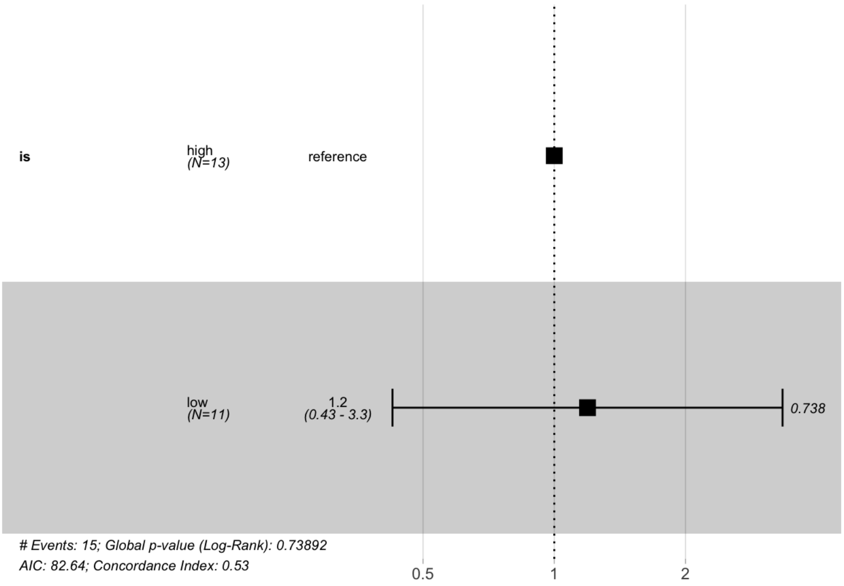

OS - Strata: IIIb

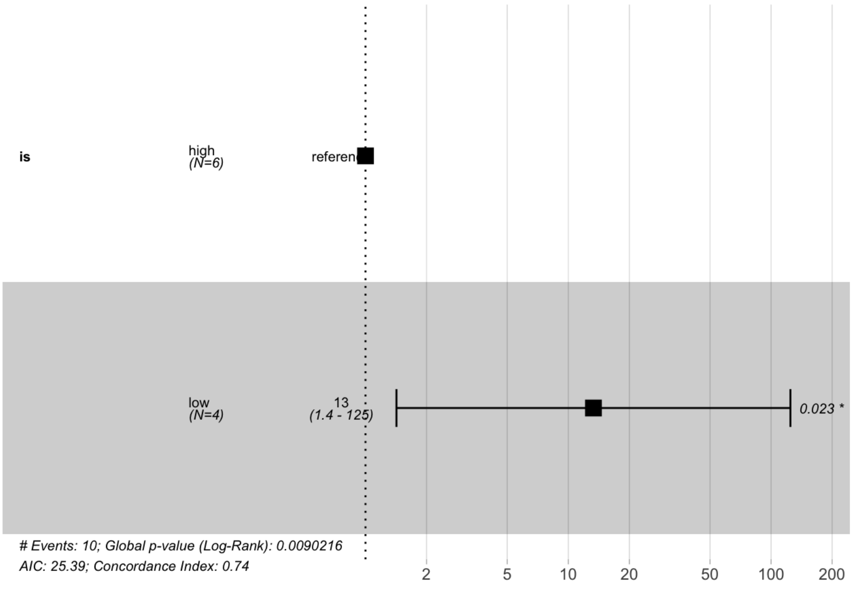

The predictive model incorporating the log-transformed average densities of CD3, CD8, CD45RO and FoxP3 can stratify patients into "high immunoscore / favourable risk" and "low immunoscore / unafavourable risk". This effect cannot be observed among AJCC IIIb patients as their survival is probably mainly driven by the presence of the nodal disease. The split-sample approach is limited by the sample size.

Conclusions

- Immunoscore is a protective factor for the three clinically meaningful outcomes PFS, CSS and OS.
- Moderate effect measure in comparison to known predictors such as AJCC stages.
- Potential to substratify patients within AJCC stages "0is/0a/I", "II" and "IIIa" into "high immunoscore / favourable risk" and low immunoscore / unfavourable risk".
- In the current setting (patients undergoing radical cystectomy for bladder cancer), it might serve as an additional tool to select patients for adjuvant therapies.
- We have currently a sample size limitation and cannot perform a sufficiently powered internal validation by a split-sample approach.
- Next steps: External validation? TURBT samples?
